# Label-Aware Entropic Distributional Contrastive Alignment for Multimodal Sentiment Analysis

**DOI:** 10.3390/e28101062

**Published:** 2026-09-26

**Authors:** Mengyao Wang, Xiuyang Meng, Chunling Wang

**Affiliations:** 1 School of Information Science and Technology, Beijing Forestry University, Beijing 100083, China; mengyao_wang@bjfu.edu.cn; 2 The Chinese University of Hong Kong, Hong Kong SAR, China; xiuyangmeng@link.cuhk.edu.hk; 3 Hebei Key Laboratory of Smart National Park, Beijing 100083, China

**Keywords:** multimodal sentiment analysis, contrastive learning, multimodal representation, optimal transport

## Abstract

Multimodal sentiment analysis integrates text, audio, and visual signals to infer affective states. However, sentiment information shared across modalities is often entangled with modality-specific variation, and existing representation learning methods do not fully exploit the relations encoded by continuous sentiment labels. Effective cross-view alignment therefore requires structured representations and a target that captures affective relations among samples. We propose a label-aware distributional contrastive alignment framework to address these requirements. Shared-private representation learning separates common sentiment information from modality-specific cues. The shared representations form audio–text and visual–text interaction views guided by a common textual reference, while the private representations retain complementary cues for prediction. Label-Aware GCA-UOT aligns the two sets of interaction views through batch-level distributional matching. Entropic unbalanced optimal transport provides a smooth, flexible coupling, and a label-aware target guides the allocation of matching mass according to paired-view correspondence, sentiment-intensity proximity, and polarity consistency. Experiments on CMU-MOSI, CMU-MOSEI, and CH-SIMS show consistent gains in fine-grained sentiment classification. Our method improves Acc-5 by 0.5–2.9 percentage points over the strongest baseline on each dataset and achieves the best or joint-best Acc-7 among the compared methods on CMU-MOSI and CMU-MOSEI.

## 1. Introduction

Multimodal sentiment analysis (MSA) aims to infer human affective states from text, audio, and visual signals. Compared with text-only analysis, MSA can use complementary cues from verbal content, acoustic prosody, and visual behavior. Benchmarks such as CMU-MOSI, CMU-MOSEI, and CH-SIMS have supported research on fine-grained sentiment prediction in English and Chinese [1,2,3,4]. Effective MSA remains challenging because modalities differ in their distributions, temporal structures, and semantic content. Text often expresses sentiment explicitly, whereas audio and visual signals can be noisier and more dependent on context. Sentiment annotations also commonly take the form of continuous scores with an ordinal interpretation. Utterances with similar scores may therefore share affective information that is lost when samples are treated independently or assigned only to discrete categories.

Existing studies address these challenges in several ways. Fusion methods model cross-modal interactions through tensor fusion, low-rank fusion, attention, or language-model adaptation [5,6,7,8]. Representation learning methods separate information shared across modalities from modality-specific information [9,10,11,12]. Text-guided methods use textual semantics to refine non-text representations and support cross-modal interaction [13,14,15,16]. These approaches improve how multimodal features are constructed and combined. Continuous sentiment prediction also requires attention to how the resulting representations are aligned with the sentiment relations among samples.

Contrastive learning organizes representation spaces by imposing relational constraints among samples or views, typically encouraging related representations to remain close while separating unrelated ones [17,18]. In MSA, contrastive objectives have been employed to improve cross-modal consistency, decompose shared and modality-specific information, and exploit sentiment-aware sample relations [12,19,20]. Despite their different relation definitions, these objectives generally express relational supervision through pairwise similarities among individual samples or views. Such pairwise formulations provide effective local constraints, but they do not explicitly represent the joint matching structure between two sets of cross-view representations.

Optimal transport (OT) addresses representation alignment at the distribution level by learning a global matching plan between two representation sets [21,22]. Entropy regularization produces smooth transport plans that can be computed efficiently with Sinkhorn-type methods [23,24,25]. Unbalanced OT further relaxes strict marginal constraints to accommodate noisy or partial correspondences [26]. GCA-UOT applies this formulation to contrastive representation learning, treating cross-view alignment as transport-plan learning [27]. Its paired-instance target specifies correspondence between two views of the same utterance but does not distinguish non-paired utterances according to their sentiment labels. This distinction matters in MSA: utterances with similar sentiment intensities may be more closely related than those with substantially different scores. Although entropic UOT can allocate mass to off-diagonal entries, this flexibility alone does not identify affectively meaningful neighbors. We therefore investigate whether incorporating continuous-label relations into the target transport distribution improves the organization of cross-view representations for fine-grained sentiment prediction.

We combine shared-private representation learning, text-guided interaction, and GCA-UOT in a framework for fine-grained multimodal sentiment prediction. Shared representations form audio–text and visual–text interaction views guided by a common textual reference, while private representations retain complementary modality-specific cues. Our methodological extension, Label-Aware GCA-UOT (LA-GCA-UOT), uses a target transport distribution derived from continuous labels to supervise alignment between the interaction views. The target combines paired-view correspondence, soft neighborhood relations based on label distance, and polarity consistency. It thus specifies the desired sentiment relations within the batch-level matching structure. The interaction and private representations are then fused for prediction.

The main contributions of this work are summarized as follows:

- We present a representation-and-alignment framework for fine-grained multimodal sentiment prediction, using shared-private representations and text-guided interaction to construct the views for LA-GCA-UOT. The framework provides the representation setting for the proposed alignment target and retains private modality cues for final prediction.
- Within this framework, we extend GCA-UOT with a continuous-label target transport distribution. Paired correspondence, sentiment-distance weighting, and polarity consistency jointly specify the desired matching structure between the interaction views.
- Experiments on CMU-MOSI, CMU-MOSEI, and CH-SIMS evaluate the framework and its design choices through external-model comparisons, structural and component ablations, and sensitivity analysis. Same-backbone objective comparisons on the English benchmarks further examine the alignment formulation. The results demonstrate consistent gains in fine-grained sentiment classification across all three benchmarks.

## 2. Related Work

### 2.1. Multimodal Sentiment Analysis

The development of contextual text representations also provides a foundation for sentiment modeling. SiAraSent compares engineered lexical and emoji features with AraBERT-based representations and fine-tuning for Arabic text sentiment analysis [28], illustrating the progression from feature engineering to contextual Transformer representations. Multimodal sentiment analysis extends this representation-learning setting by incorporating acoustic and visual information.

Effective multimodal sentiment analysis requires fusion mechanisms that integrate heterogeneous text, audio, and visual signals. Research has progressed from static fusion to explicit cross-modal interaction and refinement of the fusion process. Early methods used feature concatenation, tensor composition, or low-rank approximation to preserve joint multimodal information. Later methods introduced attention, gating, and Transformers to model dynamic dependencies across modalities. More recent approaches emphasize information preservation, representation regularization, and text-guided interaction to improve the stability and discriminative quality of fused representations [5,6,7,8,15,16,29].

Several methods illustrate this progression. TFN models joint multimodal features through tensor fusion, while LMF provides an efficient low-rank approximation [5,6]. MulT uses directional cross-modal attention to capture long-range dependencies in unaligned sequences [7]. MAG-BERT incorporates audio and visual information into pretrained language models through adaptation gates [8]. MMIM emphasizes information preservation by hierarchically maximizing mutual information between unimodal inputs and fused representations [29]. Recent text-guided methods use textual semantics to refine non-text representations, exploiting text as a reference for cross-modal sentiment interaction [15,16]. Alongside these advances in fusion, another line of research examines the internal organization of multimodal representations.

Modalities differ in how they express information, in their granularity, and in their semantic content. Fusion architecture alone therefore cannot guarantee robust, discriminative representations. Multimodal representation learning addresses this issue through three broad strategies. First, translation, cross-modal interaction, and contextual modeling reduce semantic gaps and facilitate alignment in a shared space [30,31]. Second, auxiliary tasks, self-supervised learning, and knowledge enhancement provide additional training signals to improve generalization and training stability [32]. Third, structured constraints such as shared-private decomposition separate common information from modality-specific variation, reducing entanglement and improving interpretability [9,33].

Recent work also extends disentangled representation learning to incomplete multimodal inputs. Li et al. propose DERL, which combines hybrid experts for shared/private disentanglement, multi-level collaborative reconstruction, and importance-aware fusion to address missing modalities [34]. This work highlights the role of representation decomposition in recovering useful information under incomplete observations. Whereas DERL emphasizes representation recovery under missing modalities, our framework uses continuous sentiment labels to organize the distributional alignment of shared interaction views.

These strategies share a central goal: constructing representations that capture common information while preserving differences across modalities. They can reduce semantic discrepancies and improve sentiment prediction, but they focus mainly on representation construction and fusion. Less attention has been given to assigning the resulting representations distinct roles in distributional alignment. In our framework, shared representations are refined into audio–text and visual–text interaction views for LA-GCA-UOT, while private representations pass directly to the final predictor. This design connects structured representation learning with alignment while preserving modality-specific information outside the shared alignment space.

### 2.2. Contrastive Learning

Contrastive learning brings related samples or views closer in representation space and separates unrelated ones. It has been widely studied in both self-supervised and supervised settings [17,18]. In MSA, Lin and Hu combine unimodal encoding with cross-modal prediction to improve representation consistency and robustness [35]. Lin et al. use hierarchical graphs to capture intra-modal and inter-modal relations beyond whole-utterance representations [36]. ConFEDE combines contrastive learning with feature decomposition to organize shared and modality-specific information [12]. CLGSI uses sentiment-intensity differences to select and weight positive and negative pairs, providing finer relational supervision than fixed binary assignments [20].

Beyond sentiment analysis, CodeLLM-Auth combines code stylometry with pretrained code-Transformer representations through cross-attention fusion and supervised contrastive learning for source-code authorship attribution and verification [37]. This application illustrates the use of supervised contrastive objectives to organize complementary representations around author identity, whereas sentiment-aware objectives organize relations according to affective labels.

These studies show how labels can refine contrastive supervision. Their constraints, however, are primarily expressed through pairwise or anchor-wise comparisons. They adjust individual relations without explicitly optimizing a joint matching structure between two representation sets. This distinction matters for continuous sentiment prediction, where relations between non-paired views can carry different degrees of affective relevance.

We therefore use continuous labels to construct a batch-level target transport distribution. The target retains paired correspondence while using sentiment-intensity proximity and polarity consistency to define relations between non-paired views. This integrates label-aware supervision directly into distributional contrastive alignment.

### 2.3. Optimal Transport

Optimal transport (OT) learns a joint coupling between empirical distributions and has been increasingly applied to alleviate modality heterogeneity in multimodal sentiment analysis [21]. InfoEnh employs OT-enhanced domain adaptation to map heterogeneous modalities into a common feature space [38]. AlignMamba uses an OT-based local alignment module to establish token-level cross-modal correspondences before multimodal interaction [39], while PaSE applies entropic OT to improve the semantic consistency of calibrated unimodal representations before fusion [40]. These studies demonstrate the effectiveness of transport-based modeling for multimodal representation alignment. However, their transport objectives mainly organize feature-, token-, or prototype-level correspondences and are not designed to represent graded sentiment relations between two cross-view representation sets.

Parallel to these developments, sentiment-aware contrastive methods have incorporated label distance, sentiment intensity, or polarity information into positive-pair selection and pairwise weighting [20]. Compared with fixed binary positive–negative assignments, such supervision provides a finer description of inter-sample sentiment relations. Nevertheless, these relations are still primarily imposed on individual sample pairs and therefore do not directly determine the joint matching structure between two sets of cross- view representations.

OT has also been connected with contrastive representation learning. Generalized Contrastive Alignment (GCA) provides a transport-plan-based formulation in which cross-view relations are jointly represented through a coupling, rather than being optimized only as independent pairwise similarities [27]. This perspective extends contrastive learning from isolated positive–negative relations to the relational structure between two representation sets. However, the target used in the standard paired-view setting remains primarily determined by instance correspondence, while continuous sentiment intensity and polarity have not been incorporated into the target transport structure for multimodal sentiment analysis.

These lines of research address related but distinct aspects of alignment. OT-based MSA methods mainly reduce feature-level discrepancies, sentiment-aware contrastive methods encode continuous labels through pairwise constraints, and transport-based contrastive methods largely use paired-instance targets. Our method connects these ideas by using continuous sentiment relations to define a target coupling between audio–text and visual–text interaction representations. The target combines paired correspondence, sentiment-intensity proximity, and polarity consistency, extending label supervision to the joint alignment of two representation sets.

## 3. Materials and Methods

Figure 1 shows the four stages of the proposed framework: input projection, decoupled text-guided encoding, label-aware distributional contrastive alignment, and fusion for prediction. First, modality-specific projections map text, audio, and visual inputs into a common latent space. Shared and private encoders then separate the information used for cross-modal interaction from modality-specific cues retained for prediction. TGCE constructs audio–text and visual–text interaction views from the shared representations using a common textual reference. LA-GCA-UOT aligns these views with a target distribution that encodes paired correspondence and cross-sample sentiment relations. Finally, the interaction and private representations are concatenated for sentiment prediction.

### 3.1. Task Formulation

We consider the standard multimodal sentiment analysis setting, where each utterance consists of three modalities: text, audio, and visual signals. Given a preprocessed utterance, the unimodal input sequences are denoted as $U_{t}\in R^{T_{t}\times d_{t}}$, $U_{a}\in R^{T_{a}\times d_{a}}$, and  $U_{v}\in R^{T_{v}\times d_{v}}$, where $T_{m}$ and $d_{m}$ denote the sequence length and feature dimension of modality m∈{t,a,v}, respectively.

Because the input feature dimensions and representation spaces differ across modalities, we use modality-specific projections to map each input sequence into a common latent space:

(1) $$X_{m}=\phi_{m}\left(U_{m}\right),\;X_{m}\in R^{T_{m}\times d},\;m\in \left\{t,a,v\right\},$$

where $\phi_{m}\left(\cdot \right)$ denotes the projection function for modality *m*, and *d* is the unified latent feature dimension. For notational simplicity, $X_{t}$, $X_{a}$, and $X_{v}$ are used throughout the following sections to denote the projected text, audio, and visual representations, respectively.

The goal of multimodal sentiment analysis is to predict the sentiment label or sentiment intensity score *y* from the multimodal representations:

(2) $$\hat{y}=f_{\Theta }\left(X_{t},X_{a},X_{v}\right),$$

where $f_{\Theta }\left(\cdot \right)$ denotes the proposed multimodal sentiment prediction model, and $\hat{y}$ is the predicted sentiment output.

### 3.2. Decoupled Text-Guided Representation Learning

Cross-modal interaction benefits from representations that emphasize modality-consistent sentiment semantics while preserving modality-specific complementary cues. Inspired by shared-private representation learning in multimodal sentiment analysis [9,41], we first decompose each modality into shared and private representations. The shared representations provide a semantic basis for cross-modal interaction, while the private representations retain complementary modality-specific evidence for final prediction.

Given the projected modality representation $X_{m}$, we employ a shared encoder and modality-specific private encoders to obtain the sequence-level shared and private representations:

(3) $$R_{m}^{s}=E^{s}\left(X_{m}\right),\;R_{m}^{p}=E_{m}^{p}\left(X_{m}\right),\;m\in \left\{t,a,v\right\}.$$

where $R_{m}^{s},R_{m}^{p}\in R^{T_{m}\times d}$. Here, $E^{s}\left(\cdot \right)$ denotes the shared encoder and $E_{m}^{p}\left(\cdot \right)$ denotes the private encoder for modality *m*. The shared representation $R_{m}^{s}$ provides a sequence-level semantic basis for subsequent text-guided cross-modal refinement, whereas the private representation $R_{m}^{p}$ preserves sequence-level modality-specific complementary cues.

To reduce redundant correlations between the shared and private representations, we employ an orthogonality-based decoupling constraint. For modality *m*, let ${\hat{r}}_{m,b,i}^{s}\in R^{d}$ and ${\hat{r}}_{m,b,i}^{p}\in R^{d}$ denote the normalized shared and private feature vectors of the *b*-th sample at the *i*-th temporal position, respectively. The decoupling loss is defined as

(4) $$L_{dec}=\frac{1}{|M|B}\sum_{m\in M}\sum_{b=1}^{B}\frac{1}{T_{m}}\sum_{i=1}^{T_{m}}{\left({\left({\hat{r}}_{m,b,i}^{s}\right)}^{⊤}{\hat{r}}_{m,b,i}^{p}\right)}^{2},$$

where M={t,a,v}, and *B* is the batch size. This constraint reduces overlap between the shared and private representations within each modality. The shared representations support text-guided interaction, while the private representations retain complementary modality-specific information for prediction. LA-GCA-UOT subsequently organizes cross-view sentiment relations after text-guided refinement.

Text often provides explicit sentiment information and stable contextual cues in multimodal sentiment analysis [41,42,43]. We therefore use it to guide cross-modal interaction and establish a common semantic reference for label-aware alignment. Following the directional cross-modal attention formulation in MulT [7], we use a Text-guided Cross-modal Encoder (TGCE) to refine the shared audio and visual representations. For each non-text modality m∈{a,v}, the shared representation $R_{m}^{s}$ supplies the queries, and the shared text representation $R_{t}^{s}$ supplies the keys and values. The resulting interaction representation is

(5) $$h_{mt}={\mathrm{TGCE}}_{m}\left(R_{m}^{s},R_{t}^{s}\right),\;m\in \left\{a,v\right\},$$

where ${\mathrm{TGCE}}_{m}\left(\cdot \right)$ denotes the TGCE for modality *m*. It combines text-guided cross-modal attention with self-attention refinement, using $Q=R_{m}^{s}$, $K=R_{t}^{s}$, and $V=R_{t}^{s}$. This produces the audio–text representation $h_{at}$ and the visual–text representation $h_{vt}$. Both views originate from shared representations and are refined using the same textual reference, providing a common semantic basis for label-aware transport alignment. They are used for both alignment and prediction; the private representations supply additional modality-specific cues to the final predictor.

### 3.3. Label-Aware Distributional Contrastive Alignment

Conventional contrastive learning typically aligns views through instance correspondence: the paired view of an anchor is positive, and other views receive discrete positive or negative assignments [17,18]. Such objectives have been used in MSA to reduce modality gaps and improve cross-modal consistency [12,15,19]. Continuous sentiment scores provide a richer relation between utterances. Two utterances with similar intensities may offer useful mutual supervision even when they are not views of the same instance.

Following generalized contrastive alignment, we formulate cross-view contrastive learning as distributional alignment [27]. The model infers a transport-based alignment distribution from the current representations and trains it to approach a target distribution encoding the desired matching structure. Vanilla GCA-UOT primarily uses paired-view identity to define this target. LA-GCA-UOT extends the target with soft neighborhood relations based on sentiment-label distance and a mask enforcing negative/non-negative polarity consistency. The objective thus preserves paired correspondence while incorporating the graded relations encoded by continuous sentiment labels.

#### 3.3.1. Cross-View Transport Formulation

Each utterance has two sentiment-oriented interaction views: the audio–text representation $h_{at}$ and the visual–text representation $h_{vt}$. For a mini-batch of *B* samples, the two representation sets are

(6) $$H^{at}={\left\{h_{at}^{i}\right\}}_{i=1}^{B},\;H^{vt}={\left\{h_{vt}^{i}\right\}}_{i=1}^{B}.$$

We treat $H^{at}$ and $H^{vt}$ as two cross-view supports and formulate their alignment as a transport problem. Under this formulation, the diagonal relation i=j corresponds to the paired audio–text and visual–text representations from the same utterance, while the off-diagonal relation i≠j corresponds to cross-view relations between different utterances in the same mini-batch.

After $\ell_{2}$ normalization, the cross-view transport costs are computed by cosine dissimilarity:

(7) $$C_{ij}^{at\to vt}=1-{\left({\bar{h}}_{at}^{i}\right)}^{⊤}{\bar{h}}_{vt}^{j},\;C_{ij}^{vt\to at}=1-{\left({\bar{h}}_{vt}^{i}\right)}^{⊤}{\bar{h}}_{at}^{j},$$

where ${\bar{h}}_{at}^{i}$ and ${\bar{h}}_{vt}^{j}$ denote normalized interaction representations. Let d∈{at→vt,vt→at} denote one alignment direction. Given the cost matrix $C^{d}$, the transport plan $P^{d}\in R_{+}^{B\times B}$ is estimated by an entropic unbalanced optimal transport objective:

(8) $$\begin{array}{cc} P^{d}=\arg\min_{P\geq 0}\; & \langle C^{d},P\rangle +\epsilon \sum_{i=1}^{B}\sum_{j=1}^{B}P_{ij}\left(\log P_{ij}-1\right)\\ \; & +\rho_{1}\mathrm{KL}\left(P1\;∥\;a\right)+\rho_{2}\mathrm{KL}\left(P^{⊤}1\;∥\;b\right), \end{array}$$

where P is the transport-plan variable, ϵ is the entropy regularization coefficient, a and b are uniform reference marginals, and $\rho_{1}$ and $\rho_{2}$ control the strength of marginal relaxation. Each entry $P_{ij}^{d}$ represents the amount of matching mass transported from the *i*-th source-view representation to the *j*-th target-view representation. The unbalanced formulation relaxes strict marginal matching, making the learned transport plan more tolerant of noisy or weak cross-view correspondences.

The alignment loss compares the matching distributions for each source representation, so we normalize the transport plan row by row:

(9) $$P_{ij}^{d,row}=\frac{P_{ij}^{d}}{\sum_{k=1}^{B}P_{ik}^{d}+\delta },$$

where δ is a small constant for numerical stability. The resulting $P^{d,row}$ represents the learned row-wise cross-view alignment distribution inferred from the current interaction representations.

#### 3.3.2. Label-Aware Target Transport Distribution

The learned distribution $P^{d,row}$ is inferred from representation similarities through unbalanced OT. By contrast, the target distribution $P^{tgt}$ is constructed from sentiment labels and specifies the matching structure that the learned distribution should approach.

The paired-view correspondence is defined by the following identity relation:

(10) $$M_{ij}^{pair}=I\left(i=j\right).$$

This term preserves the reliable instance-level correspondence between $h_{at}^{i}$ and $h_{vt}^{i}$. To further encode continuous sentiment relations, we define a negative/non-negative polarity mask:

(11) $$M_{ij}^{pol}=I\left[s\left(y_{i}\right)=s\left(y_{j}\right)\right],\;s\left(y\right)=I\left(y<0\right),$$

where neutral samples are grouped with non-negative samples in the current implementation. This mask prevents utterances with opposite sentiment orientations from being assigned overly large soft matching weights merely because their sentiment scores are numerically close.

Combining paired-view correspondence, label-distance affinity, and the polarity mask gives the unnormalized target relation matrix:

(12) $$T_{ij}=\alpha M_{ij}^{pair}+\beta \exp\left(-\frac{|y_{i}-y_{j}|}{\sigma }\right)M_{ij}^{pol}\left(1-M_{ij}^{pair}\right),$$

where α controls the weight of paired correspondence, β controls the contribution of soft label-aware relations, and σ sets the scale of the decay with label distance. The first term assigns a dedicated weight to paired views. The second assigns soft matching mass to non-paired samples with similar sentiment intensities and the same negative/non-negative polarity. The target therefore captures local sentiment neighborhoods in addition to one-to-one instance correspondence.

The normalized label-aware target transport distribution is obtained by row normalization:

(13) $$P_{ij}^{tgt}=\frac{T_{ij}}{\sum_{k=1}^{B}T_{ik}+\delta }.$$

This target distribution converts continuous and ordinal sentiment supervision into a cross-view distributional matching structure.

##### Variational Justification

The construction in Equation (12) admits an exact variational interpretation under three modeling assumptions: absolute label difference measures sentiment discrepancy; non-paired relations are admissible only within the same negative/non-negative group; and paired correspondence receives a distinct prior weight. Together, these assumptions encode sentiment proximity, polarity consistency, and paired-view correspondence in the target. The derivation below uses the ideal normalization with δ=0; the implemented expression includes a small numerical stabilizer.

For anchor *i*, define the admissible set

$$A_{i}=\left\{i\right\}\cup \left\{j\neq i:s\left(y_{j}\right)=s\left(y_{i}\right)\right\},\;n_{i}=\left|A_{i}\right|-1.$$

For α,β,σ>0, assign a reference distribution on this set by

$$\pi_{ij}=\left\{\begin{array}{cc} \alpha /(\alpha +\beta n_{i}), & j=i,\\ \beta /(\alpha +\beta n_{i}), & j\in A_{i}\setminus \left\{i\right\}, \end{array}\right.\;d_{ij}=\left|y_{i}-y_{j}\right|.$$

The reference distinguishes the known paired correspondence from other admissible candidates, while treating those candidates equally before their label distances are considered. Minimizing the expected absolute label distance favors samples with similar sentiment scores. KL regularization penalizes deviations from the reference distribution, preserving its distinction between paired and non-paired candidates.

**Proposition** **1** (Label-aware target as a unique variational solution)**.** *Let $\Delta (A_{i})$ denote the probability simplex supported on $A_{i}$. The unique minimizer of*

$$\min_{q_{i}\in \Delta \left(A_{i}\right)}F_{i}\left(q_{i}\right)=\sum_{j\in A_{i}}q_{ij}d_{ij}+\sigma \;\mathrm{KL}\left(q_{i}∥\pi_{i}\right)$$

*is $q_{ij}^{*}=T_{ij}/\sum_{k}T_{ik}$, with zero mass outside $A_{i}$.*

**Proof.**  Set $Z_{i}=\sum_{j\in A_{i}}\pi_{ij}\exp\left(-d_{ij}/\sigma \right)$ and $q_{ij}^{*}=\pi_{ij}\exp\left(-d_{ij}/\sigma \right)/Z_{i}$. Direct substitution gives

$$F_{i}\left(q_{i}\right)=\sigma \;\mathrm{KL}\left(q_{i}∥q_{i}^{*}\right)-\sigma \log Z_{i}.$$

The KL divergence is non-negative and vanishes only at $q_{i}=q_{i}^{*}$, proving uniqueness. Substituting the reference weights and using $d_{ii}=0$ yields precisely the normalized relation in Equation (12).    □

Thus, absolute label distance with KL regularization yields the exponential affinity, the polarity mask specifies its support, and $\left(1-M_{ij}^{pair}\right)$ avoids double counting the paired prior. The proposition provides a variational basis for the target under the stated modeling assumptions.

##### Parameter Interpretation and Boundary Cases

Writing $S_{i}=\sum_{j\in A_{i}\setminus \left\{i\right\}}\exp\left(-d_{ij}/\sigma \right)$, the ideal target satisfies

$$q_{ii}^{*}=\frac{\alpha }{\alpha +\beta S_{i}},\;\frac{q_{ij}^{*}}{q_{ii}^{*}}=\frac{\beta }{\alpha }\exp\left(-d_{ij}/\sigma \right)\;\left(j\in A_{i}\setminus \left\{i\right\}\right).$$

For a fixed σ, the ideal normalized target depends on α and β only through their ratio β/α. If α>β, the paired entry exceeds every individual off-diagonal entry, but it need not contain a majority of the total mass because neighborhood size also matters. The parameter σ controls distance selectivity relative to the label scale: smaller values suppress positive-distance neighbors more strongly, while identical-label neighbors retain their prior weight. Setting β=0, or having no admissible neighbor, recovers the paired-only target.

The resulting row-wise target supervises the representation-based UOT coupling, whose entries are learned jointly across the batch under the full training objective.

#### 3.3.3. LA-GCA-UOT Objective

The LA-GCA-UOT objective optimizes the learned cross-view transport distributions toward the label-aware target distribution. We first define the row-normalized paired-view target as

(14) $$P_{ij}^{pair}=\frac{M_{ij}^{pair}}{\sum_{k=1}^{B}M_{ik}^{pair}+\delta }.$$

Since $M^{pair}$ is an identity matrix in the cross-view formulation, $P^{pair}$ provides a strict instance-level anchor between the paired audio–text and visual–text interaction representations.

For each direction d∈{at→vt,vt→at}, we define the paired-view alignment term and the label-aware soft alignment term as

(15) $$\begin{array}{cc} L_{pair}^{d}\; & =-\frac{1}{B}\sum_{i=1}^{B}\sum_{j=1}^{B}P_{ij}^{pair}\log\left(P_{ij}^{d,row}+\delta \right),\\ L_{soft}^{d}\; & =\frac{1}{B}\sum_{i=1}^{B}\sum_{j=1}^{B}P_{ij}^{tgt}\log\frac{P_{ij}^{tgt}+\delta }{P_{ij}^{d,row}+\delta }. \end{array}$$

The paired-view term stabilizes instance-level cross-view correspondence, while the soft alignment term encourages the learned transport distribution to follow the sentiment-label-aware matching structure. The final LA-GCA-UOT loss is computed by averaging the two alignment directions:

(16) $$L_{LA}=\frac{1}{2}\sum_{d\in \{at\to vt,vt\to at\}}\left(r_{1}L_{pair}^{d}+r_{2}L_{soft}^{d}\right),$$

where $r_{1}$ and $r_{2}$ balance the strict paired-view alignment and the label-aware soft distributional alignment. Through this objective, LA-GCA-UOT extends rigid paired-view contrastive learning into a label-aware distributional alignment problem over text-guided interaction representations.

### 3.4. Prediction and Training Objective

For sentiment prediction, the model combines the text-guided interaction representations with the private representations. Temporal pooling first converts the private representation sequences into utterance-level vectors:

(17) $$h_{m}^{p}=\mathrm{Pool}\left(R_{m}^{p}\right),\;m\in \left\{t,a,v\right\},$$

where Pool(·) denotes the temporal aggregation operation.

For the *i*-th sample, the final fused representation and prediction are defined as

(18) $$\begin{array}{cc} h_{f}^{i}\; & =\left[h_{vt}^{i};h_{at}^{i};h_{t}^{p,i};h_{v}^{p,i};h_{a}^{p,i}\right],\\ {\hat{y}}_{i}\; & =f_{\mathrm{pred}}\left(h_{f}^{i}\right), \end{array}$$

where $h_{vt}^{i}$ and $h_{at}^{i}$ denote the visual–text and audio–text interaction representations, respectively, and $h_{t}^{p,i}$, $h_{v}^{p,i}$, and $h_{a}^{p,i}$ denote the temporally aggregated private representations.

We use an $L_{1}$ regression loss for continuous sentiment prediction. Auxiliary supervision on the audio and visual private branches encourages them to retain complementary modality-specific sentiment cues:

(19) $$\begin{array}{cc} L_{pred}\; & =\frac{1}{B}\sum_{i=1}^{B}\left|{\hat{y}}_{i}-y_{i}\right|,\\ L_{pri}\; & =\frac{1}{B}\sum_{i=1}^{B}\left(\ell \left(f_{a}^{p}\left(h_{a}^{p,i}\right),y_{i}\right)+\ell \left(f_{v}^{p}\left(h_{v}^{p,i}\right),y_{i}\right)\right), \end{array}$$

where $f_{a}^{p}\left(\cdot \right)$ and $f_{v}^{p}\left(\cdot \right)$ are modality-specific auxiliary prediction heads, and ℓ(·) follows the same regression loss form as $L_{pred}$. The text private representation is directly involved in the final fusion and is optimized through the main prediction objective.

The overall training objective combines sentiment prediction, private supervision, shared-private decoupling, and label-aware distributional contrastive alignment:

(20) $$L=L_{pred}+\lambda_{pri}L_{pri}+\lambda_{dec}L_{dec}+\lambda_{LA}L_{LA},$$

where $\lambda_{pri}$, $\lambda_{dec}$, and $\lambda_{LA}$ are trade-off hyperparameters. The decoupling loss regularizes the separation between shared and private representations, while the LA-GCA-UOT loss promotes label-aware distributional alignment between the audio–text and visual–text interaction representations.

## 4. Experiments

### 4.1. Experimental Setup

#### 4.1.1. Datasets

We evaluate the method on three widely used MSA benchmarks: CMU-MOSI [1], CMU-MOSEI [2], and CH-SIMS [3]. CMU-MOSI contains English utterances from online opinion videos, annotated with sentiment scores from −3 to +3. CMU-MOSEI uses a similar annotation scheme and provides a substantially larger, more diverse collection of English utterances. CH-SIMS contains Chinese utterances with fine-grained sentiment scores from −1 to +1, enabling evaluation in a different language. Every sample includes text, audio, and visual inputs. Table 1 summarizes the datasets.

For all three datasets, we use the pre-extracted text, audio, and visual features provided through the MMSA framework [32]. Modality-specific projection layers map these features into a common latent space before shared-private decomposition and text- guided interaction.

#### 4.1.2. Evaluation Metrics

For CMU-MOSI and CMU-MOSEI, we report the mean absolute error (MAE), the Pearson correlation coefficient (Corr), five-class accuracy (Acc-5), seven-class accuracy (Acc-7), binary accuracy (Acc-2), and the F1 score. Unless otherwise specified, Acc-2 and F1 exclude samples with zero sentiment labels, following common MSA evaluation protocols. For CH-SIMS, we report MAE, Acc-2, Acc-5, and F1. MAE measures the absolute error between predicted and ground-truth sentiment scores, while Corr measures their linear association. Classification metrics are computed by mapping continuous scores to discrete sentiment categories according to standard evaluation protocols. Lower MAE and higher values of the other metrics indicate better performance.

#### 4.1.3. Implementation Details

All experiments used Python 3.6.13, PyTorch 1.10.2, CUDA 11.3, and cuDNN 8.2. They were run on a workstation with a single NVIDIA GeForce RTX 3090 GPU with 24 GB of memory. Table 2 summarizes the main settings.

### 4.2. Comparison with State-of-the-Art Methods

We evaluate the proposed method on CMU-MOSI, CMU-MOSEI, and CH-SIMS against representative approaches from different lines of multimodal sentiment analysis research. Early fusion and interaction-based models include TFN [5], LMF [6], MFN [44], and MulT [7]. Representation learning and disentanglement-based methods include MISA [9], Self-MM [32], and DMD [11]. Recent approaches to text-guided representation learning, modality decoupling, and contrastive multimodal optimization include ALMT [14], MVCF [45], PriSA [19], DLF [41], MECAM [46], MFON [47], and MISA-GMC [48].

ALMT and MFON provide relevant external comparisons for the text-guided interaction and contrastive representation learning aspects of our framework. ALMT uses language-guided adaptive hyper-modality learning to suppress irrelevant or conflicting audio-visual information, while MFON combines prompt-guided modality feature optimization with inter-modal contrastive learning. We reproduce both methods on all three datasets using the same multimodal features, dataset splits, and training protocol as our method. Results for the remaining baselines are taken from their respective publications, providing broader coverage of MSA approaches. Table 3 and Table 4 report the results and identify the reproduced baselines.

Table 3 reports the results on the English benchmarks. On CMU-MOSI, our method achieves the lowest MAE of 0.708 and the highest Acc-5 of 55.4. It also shares the best Acc-7 and Acc-2 scores of 47.3 and 85.8, respectively, and achieves competitive Corr and F1 scores of 0.793 and 85.7. Relative to the published PriSA results [19], MAE decreases from 0.714 to 0.708 and Acc-5 increases from 52.5 to 55.4; Acc-7 is unchanged, while Acc-2 and F1 improve. Our method also improves all reported metrics relative to DLF [41], including a reduction in MAE from 0.731 to 0.708 and increases in Acc-5 and Acc-7 from 52.3 and 47.1 to 55.4 and 47.3. Under the controlled reproduction protocol, it outperforms ALMT on all reported metrics and MFON on all metrics except Corr. These results indicate strong fine-grained classification performance alongside competitive regression and binary classification.

On CMU-MOSEI, our method achieves the highest Acc-5, Acc-7, Acc-2, and F1 among the listed results, at 56.7, 54.8, 86.3, and 86.2, respectively. It also achieves the second-lowest MAE of 0.527 and a competitive Corr of 0.771. The corresponding classification scores for PriSA are 56.2, 54.7, 85.9, and 85.9 [19]. Our method improves all reported metrics over the reproduced ALMT and MFON baselines except Corr, where it remains competitive. It also outperforms the published DLF [41], MECAM [46], and MISA-GMC [48] results on every reported metric. These findings support the effectiveness of the framework on the larger English benchmark.

The proposed method improves most metrics relative to classical fusion methods, including TFN [5], LMF [6], MFN [44], and MulT [7]. These methods combine modalities through tensor operations, low-rank approximations, memory mechanisms, or cross-modal attention, generally without explicit shared-private separation. Our framework introduces this separation before interaction, providing a structured basis for sentiment-oriented fusion and alignment.

Compared with MISA [9], Self-MM [32], and DMD [11], our method additionally applies label-aware distributional alignment to the audio–text and visual–text interaction views. LA-GCA-UOT uses soft sentiment neighborhoods and polarity consistency to organize these views according to the relations encoded by continuous labels.

The strongest gains occur in fine-grained classification, consistent with the design of label-aware alignment. MAE measures numerical prediction error and Corr measures linear association, whereas Acc-5 and Acc-7 depend on assigning predictions to the correct sentiment-intensity intervals. Soft neighborhood relations encourage nearby sentiment scores to have related representations, while the polarity mask restricts alignment across the negative/non-negative boundary. This provides a possible explanation for the improvements in fine-grained classification. Competitive MAE on CMU-MOSI and CMU-MOSEI further indicates that these gains do not come at the cost of substantially larger numerical errors.

On CH-SIMS, our method achieves the best results on all reported metrics, with MAE of 0.405, Acc-2 of 80.4, F1 of 80.6, and Acc-5 of 42.8 (Table 4). Relative to the strongest baseline for each metric, MAE decreases from 0.426 to 0.405, Acc-2 increases from 79.0 to 80.4, F1 increases from 79.7 to 80.6, and Acc-5 increases from 40.5 to 42.8. These results extend the evidence for the framework to Chinese sentiment prediction.

The results on CH-SIMS support the applicability of the same representation-and-alignment design to the evaluated English and Chinese benchmarks. Models are trained separately for each dataset using language-appropriate pretrained text representations: English representations for CMU-MOSI and CMU-MOSEI and Chinese representations for CH-SIMS. These learned textual representations supply the semantic reference for cross-modal interaction. The central role of text is also reflected in the text-input masking results in Section 4.3.2.

Overall, the results show competitive regression performance and strong fine-grained sentiment classification across the three benchmarks. The following ablations examine how the representation and alignment components contribute to this performance.

### 4.3. Ablation Study

To evaluate the contribution of each key component across dataset scales and language settings, we conduct structural and LA-GCA-UOT component ablations on CMU-MOSI, CMU-MOSEI, and CH-SIMS. CMU-MOSI and CMU-MOSEI provide English benchmarks at different scales, while CH-SIMS extends the analysis to Chinese sentiment prediction. Within each dataset, the ablations retain the experimental settings of the complete model apart from the component being examined. CH-SIMS results are averaged over three runs using the same seeds for the complete model and all ablation variants. Additional modality-input and contrastive-objective analyses are conducted on CMU-MOSI and CMU-MOSEI. The mechanisms underlying the observed effects are discussed in Section 5.

#### 4.3.1. Effects of Structural Components

To examine the contribution of each key design, we construct variants by removing shared-private decoupling, replacing TGCE with a simplified text-conditioned alternative, or removing the Label-Aware GCA-UOT contrastive learning branch under otherwise unchanged settings. The results are shown in Table 5. These variants examine the roles of representation decoupling, TGCE-based interaction-view construction, and label-aware distributional alignment, respectively.

As shown in Table 5, the full model achieves the best MAE, Acc-5, Acc-7, and F1 and tied-best Acc-2 on CMU-MOSI, and it achieves the best or tied-best results across all reported metrics on CMU-MOSEI. On CH-SIMS, it achieves the highest Acc-5 of 42.8%. Removing decoupling, TGCE, or the contrastive learning branch reduces Acc-5 to 40.8%, 40.5%, and 40.8%, respectively. These results support the contribution of each design to fine-grained sentiment classification across the three benchmarks.

The CH-SIMS ablations reveal trade-offs across metrics. Removing decoupling or TGCE improves MAE, Acc-2, and F1 but reduces Acc-5. Removing the contrastive branch improves MAE while lowering all three classification scores. Removing both decoupling and the contrastive branch gives MAE of 0.408 and Acc-5 of 42.7%, with worse performance than the complete model on all four metrics. The smaller Acc-5 decrease under joint removal than under either individual removal suggests that the components interact. The complete framework’s clearest advantage on CH-SIMS is fine-grained classification.

#### 4.3.2. Effects of Different Features and Modalities

To analyze the contribution of each input modality within the proposed framework, we conduct modality-input masking ablations while retaining the full model architecture. For each variant, the input features of one modality are set to zero throughout training, validation, and testing, and the model is independently retrained under this condition. Training and evaluation therefore use the same input-masking condition. A related fixed-architecture ablation strategy was used in MMCL, which zeroed the corresponding modality representations [35].

Keeping the masked branches in place preserves the architecture while isolating the contribution of the corresponding input information. In addition, we remove the private representations from the final fusion to examine whether modality-specific complementary cues provide useful information beyond the interaction representations. The results are shown in Table 6.

As shown in Table 6, masking the text input and retraining causes the largest performance drop on both datasets, supporting the central contribution of textual information within the proposed text-guided framework. Masking audio or visual inputs also reduces performance despite retraining, although the decreases are smaller. These results support the contributions of all three input modalities to sentiment prediction within this framework.

Removing private representations has mixed effects on CMU-MOSEI: the variant remains competitive on several metrics but performs worse on Acc-7, Acc-2, and F1. On CMU-MOSI, performance decreases across all reported metrics. These results suggest that the interaction views capture much of the shared sentiment information, while private representations add useful affective cues, particularly on the smaller dataset.

#### 4.3.3. Component Analysis of LA-GCA-UOT

To further analyze the contribution of each component in LA-GCA-UOT, we conduct an ablation study by modifying the internal terms of the proposed objective. Specifically, we examine the explicit pairwise alignment term $L_{pair}$, the soft distributional alignment term $L_{soft}$, the label-aware relation in the target transport distribution, and the negative/non-negative polarity mask. The results are shown in Table 7.

In w/o $L_{pair}$, we set $r_{1}=0$ and retain $r_{2}$, removing the explicit paired-view loss while preserving paired correspondence in $P^{tgt}$. In w/o $L_{soft}$, we set $r_{2}=0$, leaving only explicit paired-view alignment. In w/o label-aware relation, we set β=0, removing the label-distance relations from the target; the polarity mask then has no effect. In w/o polarity mask, we retain the label-aware relations but allow them across the negative/non-negative boundary.

As shown in Table 7, the complete LA-GCA-UOT achieves the best or tied-best results on most metrics on CMU-MOSI and CMU-MOSEI. On CH-SIMS, removing $L_{pair}$, $L_{soft}$, the label-aware relation, or the polarity mask reduces Acc-5 from 42.8% to 40.0%, 40.6%, 40.2%, and 41.6%, respectively. The decreases of 2.8, 2.2, 2.6, and 1.2 percentage points, calculated from the displayed means, support the contribution of all four components to fine-grained sentiment classification in the Chinese benchmark.

Removing the paired loss, soft distributional loss, or polarity mask also worsens the other three CH-SIMS metrics. Removing the label-aware relation improves Acc-2 and F1 and leaves MAE unchanged at the reported precision of 0.405, but it substantially reduces Acc-5. Thus, the clearest benefit of label-aware relations on CH-SIMS is finer discrimination of sentiment intensity. Across the three datasets, the results support combining paired correspondence, graded label proximity, and polarity consistency.

#### 4.3.4. Comparison with Different Contrastive Objectives

We compare LA-GCA-UOT with related objectives using the same backbone and training protocol. The w/o Contrastive Learning variant removes the entire contrastive branch. Distance-aware CL uses label-distance supervision without OT-based matching, while vanilla GCA-UOT uses distributional alignment without a sentiment-aware target. Table 8 reports the results.

LA-GCA-UOT achieves the strongest overall results in Table 8. On CMU-MOSEI, it leads on every metric. On CMU-MOSI, it achieves the lowest MAE, the highest Acc-5, Acc-7, and F1, and joint-best Acc-2. These results support the complete objective over the alternatives evaluated here.

The comparisons examine two complementary aspects of LA-GCA-UOT. Distance-aware CL supplies pairwise label-distance supervision, while vanilla GCA-UOT supplies distributional transport alignment with a paired-view target. LA-GCA-UOT combines continuous-label relations with distributional matching and improves all six reported metrics over both alternatives on CMU-MOSI and CMU-MOSEI. Because the input features, representation backbone, and training protocol are shared, these results directly support the effectiveness of the proposed alignment objective.

Together, the external-model reproductions, objective comparisons, and component ablations evaluate the method at three levels. ALMT and MFON provide complete-model comparisons across the three benchmarks. The same-backbone objective comparisons assess closely related alignment alternatives, and the component ablations identify the contributions of the target and loss terms. The consistent fine-grained classification gains across these controlled comparisons support the contribution of label-aware distributional alignment to the complete framework.

#### 4.3.5. Sensitivity to Target-Distribution Hyperparameters

Following the variational characterization in Proposition 1, we empirically examine the target-distribution parameters. Under ideal row normalization, only the ratio β/α matters, so we fix α=1 as the reference scale. The parameters β and σ control the relative contribution of non-paired relations and their selectivity with respect to label distance, respectively.

We examine local sensitivity on CMU-MOSEI around the default setting (α,β,σ)=(1.0,0.1,0.5). With α=1, we vary β∈{0.05,0.1,0.2} at σ=0.5 and σ∈{0.25,0.5,1.0} at β=0.1. The two parameters are varied one at a time. Each setting is evaluated over three runs using the same random seeds as the main baseline comparisons. Table 9 reports the mean results, with the original default result included as a reference.

With σ=0.5, the default β=0.1 produces better mean results than 0.05 or 0.2 on all six metrics, favoring an intermediate weight for non-paired relations among the tested settings. With β=0.1, MAE stays within approximately 0.527–0.528 and Corr within approximately 0.771–0.772 across the tested bandwidths. The default bandwidth gives the highest classification scores, while σ=0.25 gives the highest Corr. These results support the default as a useful balance between regression and classification within the tested CMU-MOSEI range.

The parameter settings for all three datasets are listed in Table 2. We use σ=0.5 for CMU-MOSI and CMU-MOSEI, whose sentiment labels range over [−3,3], and σ=0.15 for CH-SIMS, whose labels range over [−1,1]. The smaller bandwidth on CH-SIMS is consistent with its narrower label scale, since the affinity depends on the ratio $|y_{i}-y_{j}|/\sigma$.

#### 4.3.6. Sensitivity to Training-Label Noise

To evaluate the dependence of LA-GCA-UOT on annotation reliability, we perturb all continuous training labels on CMU-MOSEI and compare the complete model with the variant without label-aware relations (β=0). For each original label $y_{i}\in \left[-3,3\right]$, we generate

$${\tilde{y}}_{i}=3\;\mathrm{clip}\;\left(\frac{y_{i}}{3}+\eta_{i},-1,1\right),\;\eta_{i}∼N\left(0,\sigma_{n}^{2}\right),$$

where the noise is sampled independently and $\sigma_{n}\in \left\{0.05,0.10\right\}$ is measured on the normalized label scale. We use $\sigma_{n}$ to distinguish the noise standard deviation from the label-affinity bandwidth σ in the target distribution. Before clipping, these settings correspond to standard deviations of 0.15 and 0.30 on the original label scale.

The corrupted labels are generated once before training and remain fixed throughout all epochs of a run. Both the main regression objective and the private-branch auxiliary regression objectives use these labels. The complete model also uses the same corrupted labels to compute label similarities and polarity masks when constructing its target transport distribution. For each seed and noise level, the complete and β=0 models use identical corrupted labels. Validation and test labels remain unchanged. The training batch size is 64 and the early stopping patience is 5. For each nonzero noise level, we report the mean over seeds 694, 3480, and 2021. Table 10 also includes the existing noise-free results under the same training settings as a reference.

At $\sigma_{n}=0.05$, the complete model retains an advantage over β=0 in regression and fine-grained classification: MAE decreases by 0.0116, Corr increases by 0.0055, and Acc-5 and Acc-7 improve by 1.43 and 1.38 percentage points, respectively. Its performance also remains close to the noise-free reference, with MAE changing from 0.527 to 0.5287 and Acc-5 from 56.7% to 56.21%. These results indicate that label-aware relations can still provide useful supervision at the lower tested noise level. The nonzero binary metrics are nearly unchanged between the two variants, so the benefit is not uniform across all metrics.

At $\sigma_{n}=0.10$, both variants perform worse than at $\sigma_{n}=0.05$. The complete model’s MAE advantage over β=0 falls to 0.0008, while Corr and the classification means slightly favor β=0. Because the variants use identical corrupted regression labels, this comparison isolates the additional contribution of label-aware relations under the same annotation noise. Their benefit is retained at the lower tested noise level but diminishes under stronger corruption, indicating a dependence on annotation reliability.

#### 4.3.7. Sensitivity to the Number of Samples Used for Relation Computation

The number of samples participating in each relation computation determines the available cross-sample neighborhood in LA-GCA-UOT. To isolate this effect, we fix the training batch size at 64 on CMU-MOSEI and vary the relation scope K∈{16,32,64}. For K=16 or 32, the same training batch is randomly partitioned into four or two disjoint groups, respectively; UOT, the target distribution, and the alignment loss are computed separately within each group. For K=64, the original full-batch relation computation is retained. Group alignment losses are averaged with weights proportional to group sample counts, preserving the overall alignment-loss weight. The training batch size and early stopping patience of 5 are unchanged, and results are summarized over seeds 694, 3480, and 2021.

Table 11 shows that K=64 gives the best mean on every reported metric. Relative to K=16, it reduces MAE from 0.532 to 0.527 and increases Acc-5 and Acc-7 by 0.7 and 0.6 percentage points, respectively. The response is not monotonic: K=32 performs worse than K=16 on MAE, Corr, Acc-5, and Acc-7 but slightly better on the binary metrics. These results favor full-batch relation computation for the evaluated configuration. Keeping the training batch size and alignment-loss weight fixed allows the comparison to focus on the number of samples used to compute relations.

#### 4.3.8. Sensitivity to Batch Neighborhood Quality

To evaluate the dependence of label-aware alignment on batch composition, we compare the complete model with its β=0 variant on CMU-MOSEI using batches rearranged to reduce effective sentiment-neighborhood mass. For sample *i* in epoch *e*, let $B_{e}\left(i\right)$ denote its batch. Following the parameter analysis of Equation (12), we measure the available off-diagonal affinity by

(21) $$S_{i}^{\left(e\right)}=\sum_{\begin{array}{c} j\in B_{e}\left(i\right)\\ j\neq i \end{array}}1\;\left[s\left(y_{j}\right)=s\left(y_{i}\right)\right]\exp\;\left(-\frac{|y_{i}-y_{j}|}{\sigma }\right),\;s\left(y\right)=1\left(y<0\right),\;\sigma =0.5.$$

We use the original CMU-MOSEI labels, preserving their values throughout the experiment. This measure excludes self-relations and sums the affinities of same-polarity neighbors, with zero labels assigned to the non-negative group. It is computed for both model variants using the same labels and batch indices, independently of the value of β.

For each seed and epoch, we first generate a random sample permutation and apply drop_last=True. This defines the used set $U_{e}$, containing 16,320 samples in 255 batches of size 64, and the six omitted samples. Candidate batch assignments are obtained by exchanging used samples across batches. Each sample in $U_{e}$ occurs exactly once, and the omitted set remains fixed. Let $S_{i,\mathrm{random}}^{\left(e\right)}$ and $S_{i,\mathrm{swap}}^{\left(e\right)}$ denote the neighborhood masses before and after the exchanges. We define

(22) $${\bar{S}}_{c}^{\left(e\right)}=\frac{1}{|U_{e}|}\sum_{i\in U_{e}}S_{i,c}^{\left(e\right)},\;c\in \left\{\mathrm{random},\mathrm{swap}\right\},\;\rho_{e}^{\mathrm{nbr}}=1-\frac{{\bar{S}}_{\mathrm{swap}}^{\left(e\right)}}{{\bar{S}}_{\mathrm{random}}^{\left(e\right)}}.$$

Thus, the reduction is measured relative to the original random batches for the same seed, epoch, and used sample set. An exchange is accepted only when it lowers the total neighborhood mass over $U_{e}$.

We evaluate two exchange policies. The targeted policy stops when the reduction reaches the prescribed 2.00–2.05% interval, subject to a maximum of $8|U_{e}|$ attempted exchanges per epoch. Its observed reductions during training were 2.000–2.005%. The fixed-budget policy performs $8|U_{e}|$ exchange attempts per epoch; the reduction is an outcome of the search.

For each policy, the complete model and the β=0 variant use identical batch indices for each matched seed and epoch. The batch size, used and omitted sample sets, and number of optimizer updates remain fixed within each epoch, with 1 update per batch and 255 updates in total. Early stopping is applied independently to each run, so total training lengths may differ. Table 12 reports the means and sample standard deviations over three seeds for each model and policy.

Under the targeted 2% policy, the complete model improves all four reported means over β=0: MAE decreases by 0.0059, and Acc-5, Acc-7, and Acc-2 increase by 0.24, 0.52, and 0.15 percentage points, respectively. Label-aware relations therefore retain predictive benefits under this controlled reduction in neighborhood mass. Under the fixed-budget policy, the MAE advantage is 0.0022, while Acc-5, Acc-7, and Acc-2 are lower by 0.20, 0.18, and 0.03 percentage points. The classification benefit is consequently absent in the reported means for this policy. Together, the matched-model comparisons show that the additional benefit of label-aware alignment depends on the batch composition induced by the exchange policy, providing empirical support for the role of available sentiment neighbors.

### 4.4. Visualization Analysis

#### 4.4.1. Visualization of LA-GCA-UOT Matching Patterns

We visualize the row-normalized transport distribution $P^{vt\to at,row}$ on CMU-MOSEI before and after alignment optimization to examine the learned cross-view matching patterns. Rows correspond to visual–text representations $h_{vt}$, and columns correspond to audio–text representations $h_{at}$. Each entry gives the transport weight from a source visual–text view to a target audio–text view. Diagonal entries link the two views of the same utterance.

Both panels use the same test mini-batch, the same label-based ordering of rows and columns, and a common color scale. Corresponding positions therefore refer to the same sample pairs, and their weights can be compared directly. Sorting by ground-truth label places utterances with similar sentiment scores near one another along both axes.

In Figure 2, weights in the initial panel are more dispersed, whereas the later panel shows more pronounced weights at paired positions and in parts of the label-adjacent regions. Under the fixed ordering and common color scale, these differences illustrate changes in the allocation of transport mass for the displayed batch. The diagonal entries correspond to paired views of the same utterance; off-diagonal entries show how the remaining mass is distributed among other samples.

The heatmaps provide a qualitative illustration of the learned coupling, complementing the prediction results and the component and objective comparisons in Section 4.3.3 and Section 4.3.4.

#### 4.4.2. Visualization of Text-Guided Interaction Representations

We use t-SNE to visualize the audio–text representations $h_{at}$ and visual–text representations $h_{vt}$ for the entire CMU-MOSEI test set. The plots before and after training use the same samples and a fixed t-SNE random state. Point colors indicate ground-truth sentiment intensity, from lower values for negative sentiment to higher values for positive sentiment.

Figure 3 shows that sentiment regions overlap considerably before training. After training, both $h_{at}$ and $h_{vt}$ exhibit clearer organization by sentiment: samples with similar intensities tend to lie closer together, and negative and positive samples are more distinguishable.

This qualitative observation is consistent with the quantitative improvements reported above. Since $h_{at}$ and $h_{vt}$ are constructed through text-guided interaction and further regularized by LA-GCA-UOT, the trained interaction space is encouraged to preserve both cross-modal correspondence and sentiment-related local structure. As t-SNE is a two-dimensional projection of high-dimensional representations and may be affected by projection hyperparameters, the visualization is used only as complementary qualitative evidence.

## 5. Discussion

### 5.1. Structured Representations for Effective Cross-Modal Interaction

The proposed framework suggests that effective multimodal sentiment learning depends on both cross-modal interaction and the structure of the representations being exchanged. Fusion methods show that richer information exchange can improve prediction [5,6,7,8]. However, when shared sentiment information is entangled with modality-specific variation, interaction may propagate irrelevant information along with affective cues. Greater interaction capacity alone does not necessarily organize representations around sentiment.

Shared-private representation learning addresses this issue by separating common semantic information from complementary modality-specific cues [9,11,33,41]. Shared representations support information exchange, while private representations preserve acoustic, visual, and textual information that is not fully captured in a common space [49]. In our framework, this decomposition determines which information reaches each stage: shared representations enter cross-modal interaction, and private representations contribute directly to prediction.

The three stages serve successive roles. Shared-private decomposition organizes information within each modality. Text-guided interaction then constructs audio–text and visual–text views from the shared representations using a common textual reference. LA-GCA-UOT aligns these views across samples. The framework thus connects within-modality decomposition, cross-modal interaction, and cross-sample relational supervision.

The structural ablations examine the roles of representation decoupling, text-guided interaction, and LA-GCA-UOT across the three datasets. The complete model achieves the highest Acc-5 in every structural comparison. On CMU-MOSI and CMU-MOSEI, the benefits extend to most regression and binary classification metrics. On CH-SIMS, retaining decoupling, TGCE, and the contrastive learning branch favors fine-grained sentiment classification, while some simplified variants achieve lower MAE or higher binary scores. The joint removal of decoupling and the contrastive learning branch produces a smaller Acc-5 decrease than either individual removal on CH-SIMS, showing that their effects depend on the remaining framework. These findings support their contribution to fine-grained discrimination while revealing dataset-dependent interactions and trade-offs among prediction metrics.

The modality ablations further clarify the contributions of text guidance and non-text inputs within the proposed framework. The largest performance degradation occurs when text is masked, consistent with its role as the semantic reference in the interaction stage. The performance decreases under audio and visual masking, even after independent retraining, further support the contributions of these modalities. The decrease caused by removing private representations also suggests that modality-private cues provide useful information beyond the shared interaction views.

The experiments assess these roles at complementary levels. Structural ablations evaluate the representation and interaction stages, while objective comparisons using the same backbone and target-component ablations evaluate the alignment extension. The fine-grained classification results support both the complete framework and the additional value of continuous-label supervision. Effects on individual metrics depend on the dataset and the components retained.

### 5.2. Label-Aware Distributional Alignment for Fine-Grained Sentiment Learning

The comparison of Distance-aware CL, vanilla GCA-UOT, and LA-GCA-UOT examines two forms of relational supervision. Distance-aware CL weights pairwise constraints by sentiment-label proximity, without learning a joint coupling between the view sets. Vanilla GCA-UOT learns a batch-level coupling but uses a paired-instance target that does not distinguish off-diagonal relations by sentiment label [27]. LA-GCA-UOT combines these properties by using label relations to define the target structure of the transport plan. Its improvements over both alternatives with the same backbone support this combination in the evaluated setting.

The label-aware target converts continuous annotations into relational supervision for the entire mini-batch. It assigns a dedicated weight to the paired audio–text and visual–text views of each utterance. For non-paired samples, weights decrease with sentiment-score distance, and the polarity mask excludes relations across the negative/non-negative boundary. The target therefore specifies both which views belong to the same utterance and how views from different utterances should be related. Continuous labels supervise the cross-view matching structure in addition to individual predictions.

Entropic unbalanced OT provides a flexible mechanism for learning this matching structure. Whereas conventional anchor-wise objectives normalize relations separately for each anchor, UOT jointly learns the entries of a batch-level coupling. Entropic regularization discourages concentration on only a few minimum-cost matches, allowing the coupling to represent both paired and weaker off-diagonal relations. It also supports efficient Sinkhorn-type optimization [23]. Marginal relaxation allows row and column masses to deviate from uniform reference marginals, reducing the need to retain weak matches merely to satisfy fixed constraints [26]. The label-aware target specifies the desired sentiment relations, while entropy regularization and marginal relaxation govern how flexibly the coupling can represent them.

The component analysis supports the complementary roles of the two loss terms. The paired-view loss reinforces correspondence between views of the same utterance, while the soft distributional loss guides the row-normalized coupling toward the label-aware target. Removing the paired-view loss weakens explicit instance-level supervision; removing the soft loss or label-aware relations reduces supervision from graded sentiment neighborhoods. The polarity mask further restricts those neighborhoods to samples with consistent polarity. Although individual metrics respond differently, the complete configuration provides the most consistent overall performance.

The Acc-5 and Acc-7 gains are consistent with the finer relational structure encoded by the target. Label-distance weights distinguish degrees of similarity among non-paired samples, and the polarity mask excludes relations across the negative/non-negative boundary. Paired-view and regression losses continue to supervise instance correspondence and continuous score prediction. On CH-SIMS, adding label-aware relations increases Acc-5 from 40.2% to 42.8%, while MAE is unchanged at the displayed precision and binary scores decrease. Sentiment-intensity discrimination is therefore the most consistent benefit, with regression and classification trade-offs varying by dataset.

More broadly, LA-GCA-UOT uses continuous labels to supervise both individual predictions and distributional alignment. Its target jointly encodes paired correspondence and graded relations among non-paired samples. Entropic regularization supports a soft coupling, and unbalanced transport accommodates variation in cross-view correspondence without fixed marginal matching. This formulation offers a basis for exploring distributional alignment in other tasks with continuous or ordered annotations.

### 5.3. Limitations and Boundary Conditions

LA-GCA-UOT depends on the reliability of the annotations used to construct its target. In standard regression, a label directly supervises the prediction for one sample. Here, it also defines relations between that sample and others in the mini-batch. Noisy or inconsistent annotations may therefore distort both prediction and relational supervision. Section 4.3.6 examines this dependence. At $\sigma_{n}=0.05$, the complete model remains close to its noise-free performance and outperforms β=0 on MAE, Corr, and fine-grained classification. At $\sigma_{n}=0.10$, its MAE advantage falls to 0.0008, and the other reported means slightly favor β=0. Stronger annotation noise thus reduces the additional benefit of label-aware relations in the tested setting.

Mini-batch composition also affects the label-aware neighborhood. Samples in densely populated sentiment regions have more nearby neighbors, whereas rare or extreme scores may have fewer. Section 4.3.7 varies the number of samples used to compute relations, and Section 4.3.8 rearranges fixed-size batches to reduce same-polarity neighborhood mass. With the targeted 2% reduction, the complete model outperforms β=0 on all four reported means. With the fixed exchange budget, its MAE advantage is smaller and the classification means slightly favor β=0. These results link the value of relational supervision to the available batch neighborhoods. Future work could examine label imbalance by explicitly controlling the frequencies of sentiment groups within batches.

The polarity mask separates negative samples from non-negative samples, including neutral utterances. Graded polarity weights could provide a more flexible treatment of expressions near the neutral boundary.

The text-guided interaction mechanism relies on the quality of the textual semantic reference. Future work will investigate reliability-aware interaction for ambiguous or sarcastic expressions.

## 6. Conclusions

We present a framework for fine-grained multimodal sentiment prediction that combines shared-private representation learning, text-guided interaction, and GCA-UOT. Shared representations form audio–text and visual–text interaction views, while private representations retain complementary cues for prediction. The methodological extension is a target transport distribution derived from continuous labels. By combining paired correspondence, sentiment-intensity proximity, and polarity consistency, this target introduces graded sentiment relations into cross-view distributional alignment.

Experiments on CMU-MOSI, CMU-MOSEI, and CH-SIMS support the effectiveness of the framework, with the most consistent gains in fine-grained sentiment classification. Ablations show the contributions of representation decomposition, TGCE, private cues, label-aware relations, and polarity masking, while also revealing dataset-dependent trade-offs across metrics. Visualizations provide complementary evidence of more structured cross-view matching and clearer organization by sentiment. Future work will investigate reliability-aware alignment by estimating the confidence of the audio–text and visual–text views and incorporating it into the transport coupling. The aim is to reduce the influence of noisy or conflicting modality information under imperfect input conditions. 

## Figures and Tables

**Figure 1:**
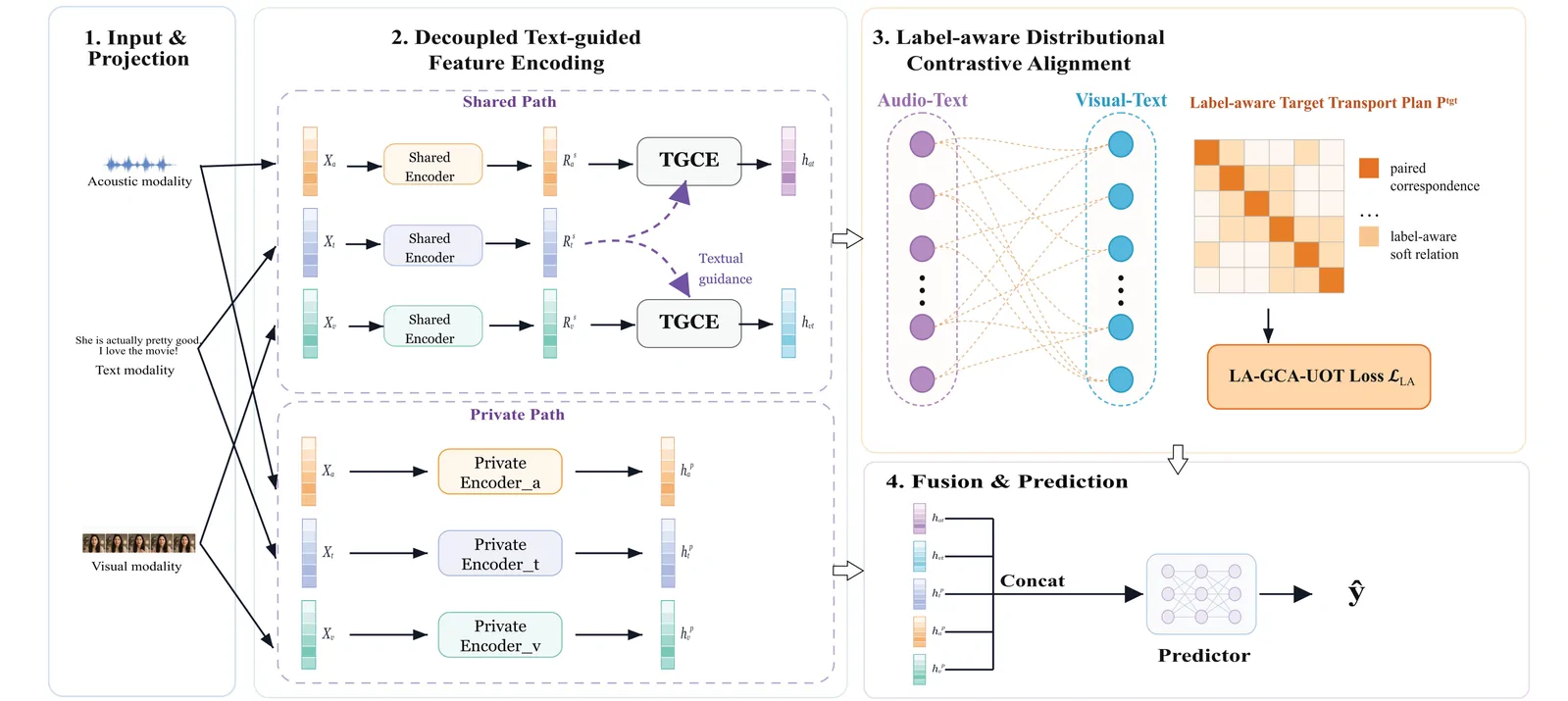
Overall framework of the proposed method.

**Figure 2:**
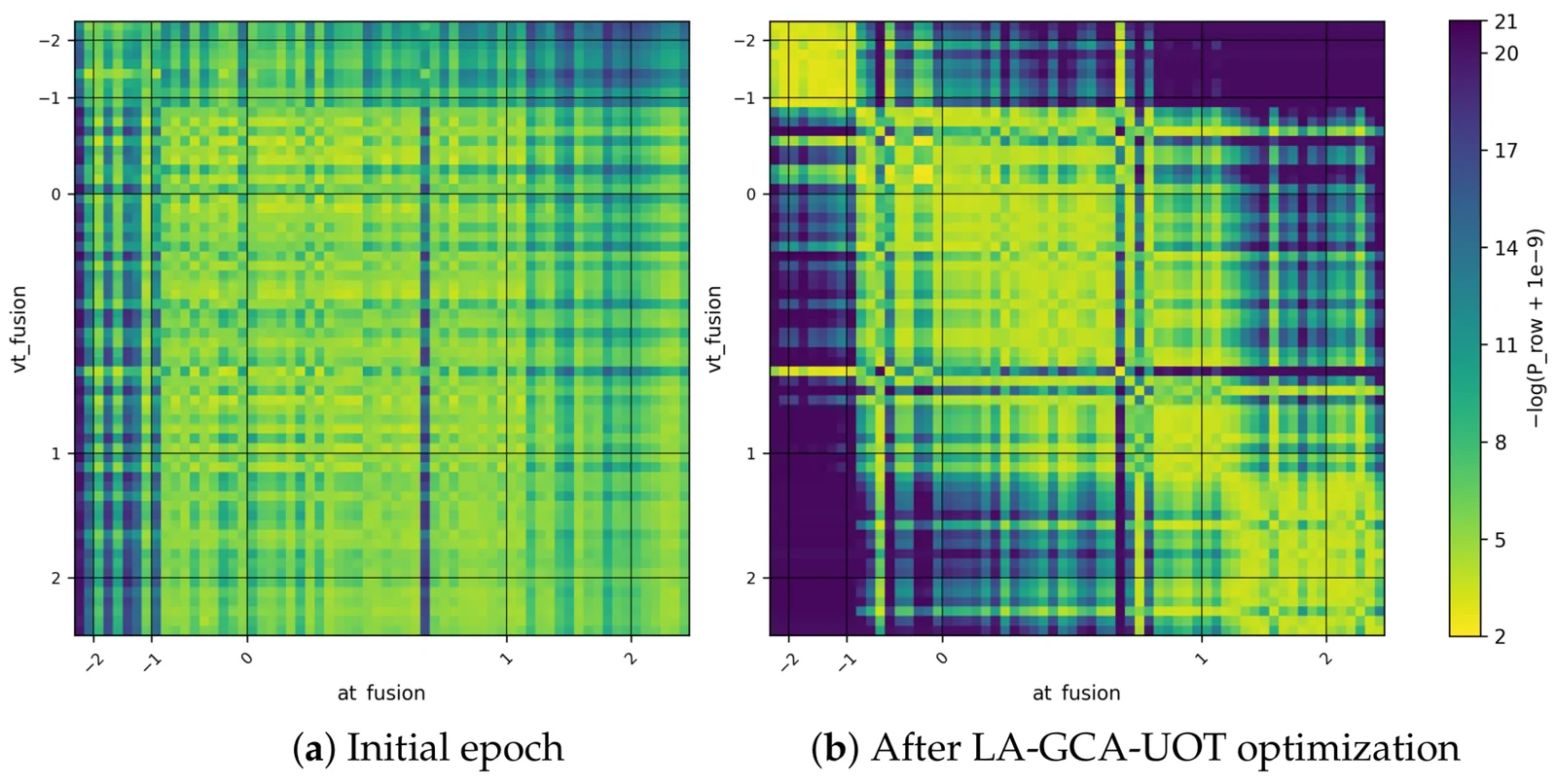
Row-normalized cross-view transport distributions $P^{vt\to at,row}$ on CMU-MOSEI at the initial epoch and after optimization. Rows denote visual–text views and columns denote audio–text views. Both panels use the same samples, the same ordering by ground-truth label on both axes, and a common color scale; brighter entries indicate larger weights. Diagonal entries represent paired views of the same utterance. Label sorting places similar-label samples near one another for display; corresponding positions in the two panels show changes in transport mass under this fixed ordering.

**Figure 3:**
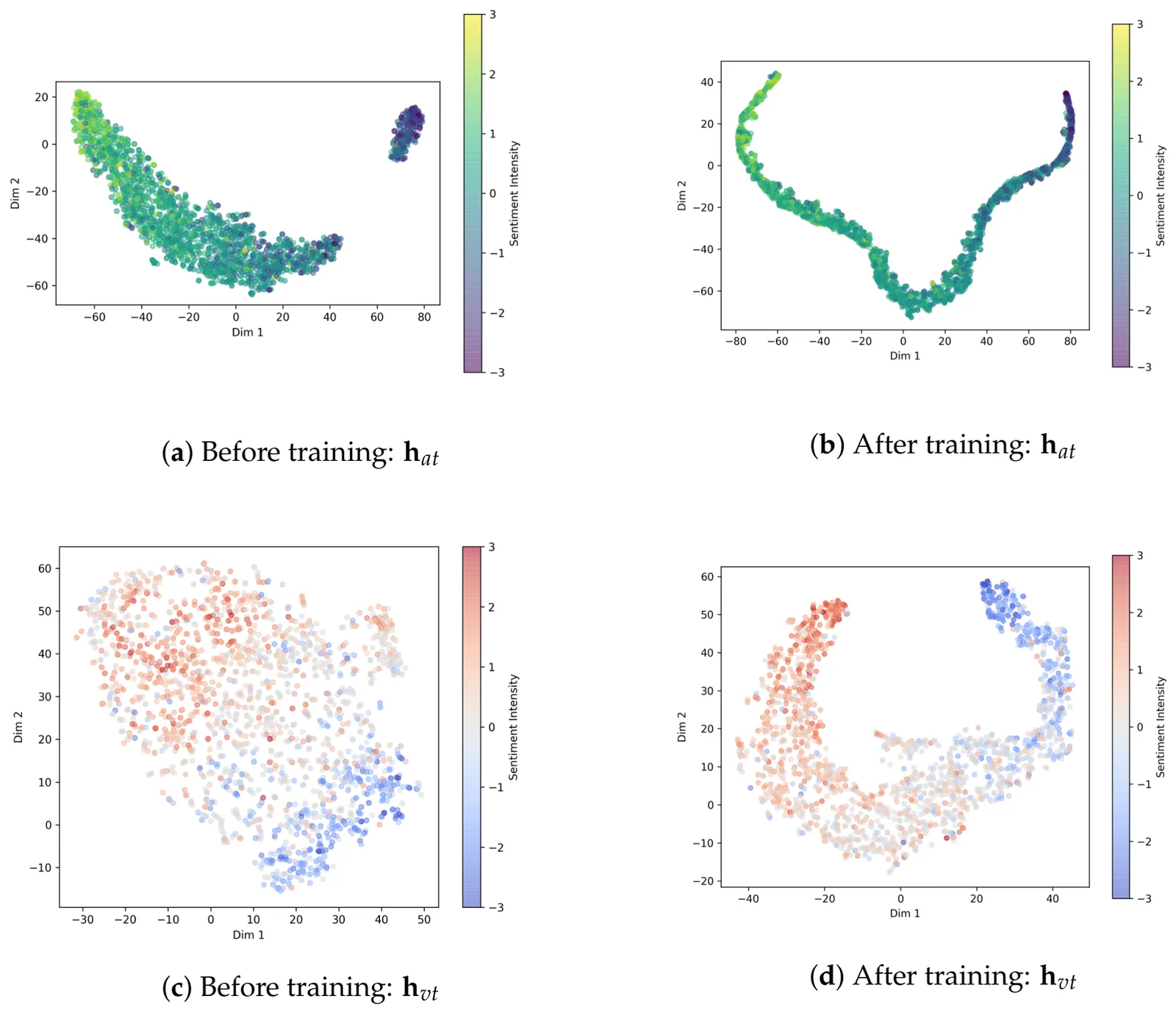
t-SNE visualization of the text-guided interaction representations on CMU-MOSEI. All four panels use the entire test set and a fixed t-SNE random state: (**a**,**b**) show the audio–text interaction representation $h_{at}$, while (**c**,**d**) show the visual–text interaction representation $h_{vt}$. Colors indicate ground-truth sentiment intensity.

**Table 1:** Statistics of the datasets used in our experiments.

Dataset	Train	Valid	Test	Language	Label Range
CMU-MOSI	1284	229	686	English	[−3, 3]
CMU-MOSEI	16,326	1871	4659	English	[−3, 3]
CH-SIMS	1368	456	457	Chinese	[−1, 1]

**Table 2:** Main hyperparameter settings of the proposed model.

Hyperparameter	CMU-MOSI	CMU-MOSEI	CH-SIMS
Batch size	16	64	16
Early stopping patience	5	5	5
Optimizer	Adam	Adam	Adam
Task loss	L1Loss	L1Loss	L1Loss
Learning rate	$1\times 10^{-4}$	$1\times 10^{-4}$	$1\times 10^{-4}$
Text encoder	bert-base-uncased	bert-base-uncased	bert-base-chinese
Hidden dimension	128	128	128
Number of attention heads	4	4	4
Text-guided cross-modal layers	1	1	1
Self-attention refinement layers	3	3	3
Modality-private encoder layers	3	3	3
Dropout rate	0.1	0.1	0.1
Decoupling loss weight $\lambda_{\mathrm{dec}}$	0.01	0.1	0.01
LA-GCA-UOT loss weight $\lambda_{\mathrm{LA}}$	0.01	0.01	0.01
Entropy regularization coefficient ϵ	0.05	0.05	0.05
$r_{1},r_{2}$	0.1, 0.05	1.0, 0.5	0.1, 0.05
α,β,σ	1.0, 0.1, 0.5	1.0, 0.1, 0.5	1.0, 0.1, 0.15
Negative/non-negative polarity mask	Enabled	Enabled	Enabled
Marginal relaxation coefficients $\left(\rho_{1},\rho_{2}\right)$	$1\times 10^{-4},\;1\times 10^{-4}$	$1\times 10^{-4},\;1\times 10^{-4}$	$1\times 10^{-4},\;1\times 10^{-4}$

**Table 3:** Comparison with state-of-the-art methods on CMU-MOSI and CMU-MOSEI. “-” indicates that the result is not reported in the original paper. Results of our method and the reproduced ALMT and MFON baselines are reported as mean ± standard deviation over three random seeds; classification means are percentages, and their standard deviations are in percentage points. The best reported mean for each metric is highlighted in bold, and the second-best mean is underlined. Tied second-best results are all underlined. ↑ indicates that higher values are better, whereas ↓ indicates that lower values are better.

Method	CMU-MOSI						CMU-MOSEI					
	MAE ↓	Corr ↑	Acc-5 ↑	Acc-7 ↑	Acc-2 ↑	F1 ↑	MAE ↓	Corr ↑	Acc-5 ↑	Acc-7 ↑	Acc-2 ↑	F1 ↑
TFN [5]	0.901	0.698	39.4	34.9	80.1	80.1	0.593	0.700	53.1	50.2	82.5	82.1
LMF [6]	0.917	0.695	38.1	33.2	82.5	82.4	0.623	0.677	52.9	48.0	82.0	82.1
MFN [44]	0.927	0.670	40.5	35.8	78.9	78.9	0.575	0.718	52.8	51.3	82.9	82.9
MulT [7]	0.871	0.698	42.7	40.0	83.0	82.8	0.580	0.703	54.2	51.8	82.5	82.3
MISA [9]	0.783	0.776	47.1	42.3	83.4	83.6	0.555	0.756	53.6	52.2	85.5	85.3
MAG-BERT [8]	0.790	0.766	-	41.4	83.5	83.6	0.543	0.755	-	52.7	84.8	84.7
Self-MM [32]	0.712	0.795	-	45.8	84.9	84.9	0.529	0.767	-	53.5	84.9	84.9
DMD [11]	0.752	-	-	46.1	83.2	83.3	0.543	-	-	52.8	84.6	84.6
ALMT ^‡^ [14]	0.762 ± 0.0334	0.785 ± 0.0042	50.3 ± 2.51	43.6 ± 1.88	83.3 ± 1.38	83.4 ± 1.33	0.538 ± 0.0079	0.771 ± 0.0020	55.0 ± 0.78	53.4 ± 0.73	85.5 ± 0.76	85.5 ± 0.64
MVCF [45]	0.718	0.790	-	45.5	85.7	85.6	0.543	0.765	-	52.8	85.5	85.4
PriSA [19]	0.714	0.792	52.5	**47.3**	85.5	85.5	**0.523**	0.772	56.2	54.7	85.9	85.9
DLF [41]	0.731	0.781	52.3	47.1	85.1	85.0	0.536	0.764	55.7	53.9	85.4	85.3
MECAM [46]	0.715	0.782	-	46.6	**85.8**	**85.8**	0.547	0.748	-	52.3	85.2	84.8
MFON ^‡^ [47]	0.713 ± 0.0043	**0.798** ± 0.0024	51.1 ± 1.20	45.3 ± 1.14	85.3 ± 0.26	85.3 ± 0.25	0.541 ± 0.0053	**0.773** ± 0.0055	54.8 ± 0.10	52.9 ± 0.37	85.8 ± 0.18	85.8 ± 0.17
MISA-GMC [48]	0.712	0.795	-	45.9	85.2	85.1	0.560	0.756	-	51.6	85.5	85.3
Ours	**0.708** ± 0.0019	0.793 ± 0.0007	**55.4** ± 2.19	**47.3** ± 1.98	**85.8** ± 0.42	85.7 ± 0.57	0.527 ± 0.0038	0.771 ± 0.0017	**56.7** ± 0.17	**54.8** ± 0.23	**86.3** ± 0.10	**86.2** ± 0.10

Note: Results marked with ^‡^ are reproduced using the same multimodal features, dataset splits, and training protocol as our method. The remaining baseline results are taken from the corresponding references.

**Table 4:** Comparison with state-of-the-art methods on CH-SIMS. “-” indicates that the result is not reported in the original paper. Results of our method and the reproduced ALMT and MFON baselines are the mean ± standard deviation over three random seeds; classification means are percentages, and their standard deviations are in percentage points. The best reported mean for each metric is highlighted in bold. ↑ indicates that higher values are better, whereas ↓ indicates that lower values are better.

Model	MAE ↓	Acc-2 ↑	F1 ↑	Acc-5 ↑
LMF [6]	0.441	77.8	77.9	40.5
TFN [5]	0.432	78.4	78.6	39.3
MFN [44]	0.437	76.4	76.2	38.3
MulT [7]	0.453	78.6	79.7	37.9
MISA [9]	0.452	74.2	74.6	36.4
ALMT ^‡^ [14]	0.434 ± 0.0042	77.2 ± 1.34	77.1 ± 1.21	38.9 ± 1.03
MFON ^‡^ [47]	0.454 ± 0.0157	76.7 ± 0.82	76.9 ± 0.37	32.9 ± 4.45
MVCF [45]	0.426	79.0	78.5	-
Ours	**0.405** ± 0.0049	**80.4** ± 0.47	**80.6** ± 0.66	**42.8** ± 0.27

Note: Results marked with ^‡^ are reproduced using the same multimodal features, dataset splits, and training protocol as our method. The remaining baseline results are taken from the corresponding references.

**Table 5:** Structural ablation study on CMU-MOSI, CMU-MOSEI, and CH-SIMS. CH-SIMS entries are the means over three matched-seed runs. The best result for each metric within each dataset is highlighted in bold, including ties at the displayed precision. ↑ indicates that higher values are better, whereas ↓ indicates that lower values are better.

(a) CMU-MOSI and CMU-MOSEI												
Variant	CMU-MOSI						CMU-MOSEI					
	MAE ↓	Corr ↑	Acc-5 ↑	Acc-7 ↑	Acc-2 ↑	F1 ↑	MAE ↓	Corr ↑	Acc-5 ↑	Acc-7 ↑	Acc-2 ↑	F1 ↑
Ours w/o Decoupling & CL Branch	0.743	0.792	51.6	44.0	84.2	84.3	0.531	0.769	55.9	54.0	85.7	85.7
Ours w/o CL Branch	0.719	**0.795**	53.4	45.1	**85.8**	85.6	0.531	0.767	56.1	54.3	85.5	85.3
Ours w/o Decoupling	0.729	0.785	52.4	45.2	84.6	84.5	0.531	**0.771**	56.0	54.2	86.1	86.1
Ours w/o TGCE	0.769	0.787	49.7	43.2	84.2	84.2	0.530	0.769	55.7	54.1	85.8	85.6
Ours	**0.708**	0.793	**55.4**	**47.3**	**85.8**	**85.7**	**0.527**	**0.771**	**56.7**	**54.8**	**86.3**	**86.2**
**(b) CH-SIMS**												
**Variant**	**MAE ↓**			**Acc-2 ↑**			**F1 ↑**			**Acc-5 ↑**		
Ours w/o Decoupling & CL Branch	0.408			80.2			80.4			42.7		
Ours w/o CL Branch	0.401			80.1			80.3			40.8		
Ours w/o Decoupling	0.401			**81.4**			**81.7**			40.8		
Ours w/o TGCE	**0.400**			80.9			81.1			40.5		
Ours	0.405			80.4			80.6			**42.8**		

Note: *Ours w/o Decoupling & CL Branch* removes both shared-private decoupling and the entire Label-Aware GCA-UOT contrastive learning branch. *Ours w/o CL Branch* removes the entire Label-Aware GCA-UOT contrastive learning branch rather than only its label-aware component. *Ours w/o TGCE* replaces the cross-attention operation in TGCE with a global text-conditioned projection, followed by the same number of self-attention layers as in the full model.

**Table 6:** Ablation study on input modalities and private representations on CMU-MOSI and CMU-MOSEI. Each input-masked variant is independently retrained with the specified modality input set to zero during training, validation, and testing, while retaining the full architecture. The best result for each metric within each dataset is highlighted in bold, including ties. ↑ indicates that higher values are better, whereas ↓ indicates that lower values are better.

Variant	CMU-MOSI						CMU-MOSEI					
	MAE ↓	Corr ↑	Acc-5 ↑	Acc-7 ↑	Acc-2 ↑	F1 ↑	MAE ↓	Corr ↑	Acc-5 ↑	Acc-7 ↑	Acc-2 ↑	F1 ↑
Text Input Masked	1.348	0.209	17.8	17.7	60.5	60.6	0.804	0.262	42.4	42.4	66.6	63.7
Visual Input Masked	0.760	0.782	50.1	42.2	83.2	83.2	0.537	0.763	55.8	54.1	84.9	85.0
Audio Input Masked	0.731	0.791	51.8	44.6	84.9	84.9	0.530	0.768	56.6	54.6	85.9	85.9
w/o Private Representations	0.759	0.781	49.1	42.7	83.7	83.9	**0.526**	**0.772**	**56.7**	54.7	85.9	85.7
Full Model	**0.708**	**0.793**	**55.4**	**47.3**	**85.8**	**85.7**	0.527	0.771	**56.7**	**54.8**	**86.3**	**86.2**

**Table 7:** Component analysis of LA-GCA-UOT on CMU-MOSI, CMU-MOSEI, and CH-SIMS. CH-SIMS entries are the means over three matched-seed runs. The best result for each metric within each dataset is highlighted in bold, including ties at the displayed precision. ↑ indicates that higher values are better, whereas ↓ indicates that lower values are better.

(a) CMU-MOSI and CMU-MOSEI												
Variant	CMU-MOSI						CMU-MOSEI					
	MAE ↓	Corr ↑	Acc-5 ↑	Acc-7 ↑	Acc-2 ↑	F1 ↑	MAE ↓	Corr ↑	Acc-5 ↑	Acc-7 ↑	Acc-2 ↑	F1 ↑
w/o $L_{pair}$	0.746	**0.793**	49.0	43.1	84.9	84.7	0.534	**0.772**	55.6	53.6	85.8	85.5
w/o $L_{soft}$	0.735	0.789	51.9	44.5	84.7	84.7	0.534	0.765	56.1	54.3	84.9	85.0
w/o label-aware relation	0.736	0.788	51.4	44.4	84.0	84.3	0.531	0.766	56.0	54.3	85.4	85.2
w/o polarity mask	0.744	0.786	51.5	44.8	85.0	84.9	**0.526**	0.770	56.6	**54.8**	85.6	85.6
LA-GCA-UOT	**0.708**	**0.793**	**55.4**	**47.3**	**85.8**	**85.7**	0.527	0.771	**56.7**	**54.8**	**86.3**	**86.2**
**(b) CH-SIMS**												
**Variant**	**MAE ↓**			**Acc-2 ↑**			**F1 ↑**			**Acc-5 ↑**		
w/o $L_{pair}$	0.411			79.8			80.0			40.0		
w/o $L_{soft}$	0.408			80.2			80.5			40.6		
w/o label-aware relation	**0.405**			**80.8**			**81.0**			40.2		
w/o polarity mask	0.406			80.3			80.5			41.6		
LA-GCA-UOT	**0.405**			80.4			80.6			**42.8**		

**Table 8:** Comparison with different contrastive objectives on CMU-MOSI and CMU-MOSEI. The best result for each metric within each dataset is highlighted in bold, including ties. ↑ indicates that higher values are better, whereas ↓ indicates that lower values are better.

Objective	CMU-MOSI						CMU-MOSEI					
	MAE ↓	Corr ↑	Acc-5 ↑	Acc-7 ↑	Acc-2 ↑	F1 ↑	MAE ↓	Corr ↑	Acc-5 ↑	Acc-7 ↑	Acc-2 ↑	F1 ↑
w/o Contrastive Learning	0.719	**0.795**	53.4	45.1	**85.8**	85.6	0.531	0.767	56.1	54.3	85.5	85.3
Distance-aware CL	0.733	0.789	50.9	43.6	85.1	85.0	0.529	0.770	56.1	54.2	85.9	85.7
Vanilla GCA-UOT	0.773	0.781	50.9	42.9	84.6	84.5	0.530	0.768	56.2	54.5	85.1	85.1
LA-GCA-UOT	**0.708**	0.793	**55.4**	**47.3**	**85.8**	**85.7**	**0.527**	**0.771**	**56.7**	**54.8**	**86.3**	**86.2**

**Table 9:** Local sensitivity to target-distribution hyperparameters on CMU-MOSEI with α=1. Entries are the means over three runs using the same random seeds as the main baseline comparisons; classification metrics are percentages. The default row reproduces the main-result reference at its original precision. ↑ indicates that higher values are better, whereas ↓ indicates that lower values are better.

β	σ	MAE ↓	Corr ↑	Acc-5 ↑	Acc-7 ↑	Acc-2 ↑	F1 ↑
0.05	0.5	0.5343	0.7675	55.88	54.05	85.58	85.44
0.1	0.5 (default)	0.527	0.771	56.7	54.8	86.3	86.2
0.2	0.5	0.5311	0.7673	56.21	54.45	86.06	85.94
0.1	0.25	0.5276	0.7718	56.44	54.53	85.69	85.58
0.1	1.0	0.5272	0.7711	56.19	54.35	85.87	85.72

**Table 10:** Sensitivity to training-label noise on CMU-MOSEI. The noise standard deviation $\sigma_{n}$ is defined on labels normalized to [−1, 1]. Entries report mean performance. Nonzero-noise results are averaged over three seeds; the noise-free rows reproduce the means in Table 7. Acc-2 and F1 exclude zero-label samples, and all classification metrics are percentages. Bold values indicate the better mean within each noise level. ↑ indicates that higher values are better, whereas ↓ indicates that lower values are better.

$\sigma_{n}$	Model	MAE ↓	Corr ↑	Acc-5 ↑	Acc-7 ↑	Acc-2 ↑	F1 ↑
0	LA-GCA-UOT	**0.527**	**0.771**	**56.7**	**54.8**	**86.3**	**86.2**
	β=0	0.531	0.766	56.0	54.3	85.4	85.2
0.05	LA-GCA-UOT	0.5287	0.7702	56.21	54.40	86.00	85.85
	β=0	0.5403	0.7647	54.78	53.02	85.96	85.91
0.10	LA-GCA-UOT	0.5475	0.7553	54.98	53.37	85.42	85.43
	β=0	0.5483	0.7561	55.04	53.42	85.55	85.54

**Table 11:** Sensitivity to relation computation scope on CMU-MOSEI with a fixed training batch size of 64. Results are the means ± sample standard deviations over three seeds. Acc-2 and F1 exclude zero-label samples, and all classification metrics are percentages. The best mean for each metric is highlighted in bold. ↑ indicates that higher values are better, whereas ↓ indicates that lower values are better.

Scope *K*	MAE ↓	Corr ↑	Acc-5 ↑	Acc-7 ↑	Acc-2 ↑	F1 ↑
16	0.532±0.007	0.767±0.003	56.0±0.4	54.2±0.6	85.8±0.2	85.6±0.2
32	0.537±0.007	0.761±0.007	55.4±1.2	53.7±1.0	85.9±0.3	85.8±0.3
64	0.527±0.004	0.771±0.002	56.7±0.2	54.8±0.2	86.3±0.1	86.2±0.1

**Table 12:** Sensitivity to batch neighborhood quality on CMU-MOSEI. Results are the means ± sample standard deviations over three seeds; classification metrics are percentages. Bold indicates the better mean within each exchange policy. The targeted policy achieved a 2.000–2.005% reduction during training. ↑ indicates that higher values are better, whereas ↓ indicates that lower values are better.

Model	MAE ↓	Acc-5 ↑	Acc-7 ↑	Acc-2 ↑
Targeted 2% neighborhood-mass reduction				
LA-GCA-UOT	0.5277±0.0014	56.10±0.32	54.47±0.33	85.84±0.06
β=0	0.5336±0.0028	55.86±0.21	53.95±0.28	85.69±0.48
Fixed budget of $8|U_{e}|$ exchange attempts per epoch				
LA-GCA-UOT	0.5317±0.0041	55.64±0.23	53.94±0.28	85.75±0.04
β=0	0.5339±0.0040	55.84±0.28	54.12±0.18	85.78±0.35

## Data Availability

The CMU-MOSI, CMU-MOSEI, and CH-SIMS datasets and the processed multimodal features analyzed in this study are publicly available through the MMSA repository at https://github.com/thuiar/MMSA (accessed on 30 July 2026). No new datasets were created in this study.

## References

[1] Zadeh A., Zellers R., Pincus E., Morency L.P. (2016). MOSI: Multimodal Corpus of Sentiment Intensity and Subjectivity Analysis in Online Opinion Videos. arXiv.

[2] Bagher Zadeh A., Liang P.P., Poria S., Cambria E., Morency L.P. (2018). Multimodal Language Analysis in the Wild: CMU-MOSEI Dataset and Interpretable Dynamic Fusion Graph. Proceedings of the 56th Annual Meeting of the Association for Computational Linguistics (Volume 1: Long Papers).

[3] Yu W., Xu H., Meng F., Zhu Y., Ma Y., Wu J., Zou J., Yang K. (2020). CH-SIMS: A Chinese Multimodal Sentiment Analysis Dataset with Fine-grained Annotation of Modality. Proceedings of the 58th Annual Meeting of the Association for Computational Linguistics.

[4] Soleymani M., Garcia D., Jou B., Schuller B., Chang S.F., Pantic M. (2017). A Survey of Multimodal Sentiment Analysis. Image Vis. Comput..

[5] Zadeh A., Chen M., Poria S., Cambria E., Morency L.P. (2017). Tensor Fusion Network for Multimodal Sentiment Analysis. Proceedings of the 2017 Conference on Empirical Methods in Natural Language Processing.

[6] Liu Z., Shen Y., Lakshminarasimhan V.B., Liang P.P., Bagher Zadeh A., Morency L.P. (2018). Efficient Low-rank Multimodal Fusion With Modality-Specific Factors. Proceedings of the 56th Annual Meeting of the Association for Computational Linguistics (Volume 1: Long Papers).

[7] Tsai Y.H.H., Bai S., Liang P.P., Kolter J.Z., Morency L.P., Salakhutdinov R. (2019). Multimodal Transformer for Unaligned Multimodal Language Sequences. Proceedings of the 57th Annual Meeting of the Association for Computational Linguistics.

[8] Rahman W., Hasan M.K., Lee S., Bagher Zadeh A., Mao C., Morency L.P., Hoque E. (2020). Integrating Multimodal Information in Large Pretrained Transformers. Proceedings of the 58th Annual Meeting of the Association for Computational Linguistics.

[9] Hazarika D., Zimmermann R., Poria S. (2020). MISA: Modality-Invariant and -Specific Representations for Multimodal Sentiment Analysis. Proceedings of the 28th ACM International Conference on Multimedia.

[10] Yang D., Huang S., Kuang H., Du Y., Zhang L. (2022). Disentangled Representation Learning for Multimodal Emotion Recognition. Proceedings of the 30th ACM International Conference on Multimedia.

[11] Li Y., Wang Y., Cui Z. Decoupled Multimodal Distilling for Emotion Recognition. Proceedings of the IEEE/CVF Conference on Computer Vision and Pattern Recognition.

[12] Yang J., Yu Y., Niu D., Guo W., Xu Y. (2023). ConFEDE: Contrastive Feature Decomposition for Multimodal Sentiment Analysis. Proceedings of the 61st Annual Meeting of the Association for Computational Linguistics (Volume 1: Long Papers).

[13] Wang D., Guo X., Tian Y.m., Liu J., He L., Luo X. (2023). TETFN: A Text Enhanced Transformer Fusion Network for Multimodal Sentiment Analysis. Pattern Recognit..

[14] Zhang H., Wang Y., Yin G., Liu K., Liu Y., Yu T. Learning Language-guided Adaptive Hyper-modality Representation for Multimodal Sentiment Analysis. Proceedings of the 2023 Conference on Empirical Methods in Natural Language Processing.

[15] Chen J., Song S., Tan Y., Xia H. (2025). TEMSA: Text Enhanced Modal Representation Learning for Multimodal Sentiment Analysis. Comput. Vis. Image Underst..

[16] Su Y., Zhao X. (2025). Hierarchical Text-Guided Refinement Network for Multimodal Sentiment Analysis. Entropy.

[17] van den Oord A., Li Y., Vinyals O. (2018). Representation Learning with Contrastive Predictive Coding. arXiv.

[18] Khosla P., Teterwak P., Wang C., Sarna A., Tian Y., Isola P., Maschinot A., Liu C., Krishnan D. (2020). Supervised Contrastive Learning. Adv. Neural Inf. Process. Syst..

[19] Ma F., Zhang Y., Sun X. Multimodal Sentiment Analysis with Preferential Fusion and Distance-Aware Contrastive Learning. Proceedings of the 2023 IEEE International Conference on Multimedia and Expo.

[20] Yang Y., Dong X., Qiang Y. (2024). CLGSI: A Multimodal Sentiment Analysis Framework Based on Contrastive Learning Guided by Sentiment Intensity. Proceedings of the Findings of the Association for Computational Linguistics: NAACL 2024.

[21] Peyré G., Cuturi M. (2019). Computational Optimal Transport. Found. Trends Mach. Learn..

[22] Wang S., Stavrou P.A., Skoglund M. (2022). Generalizations of Talagrand Inequality for Sinkhorn Distance Using Entropy Power Inequality. Entropy.

[23] Cuturi M. (2013). Sinkhorn Distances: Lightspeed Computation of Optimal Transport. Adv. Neural Inf. Process. Syst..

[24] Tong Q., Kobayashi K. (2021). Entropy-Regularized Optimal Transport on Multivariate Normal and q-Normal Distributions. Entropy.

[25] Wang J., Wang P., Shafto P. (2023). Efficient Discretization of Optimal Transport. Entropy.

[26] Chizat L., Peyré G., Schmitzer B., Vialard F.X. (2018). Scaling Algorithms for Unbalanced Optimal Transport Problems. Math. Comput..

[27] Chen Z., Lin C.H., Liu R., Xiao J., Dyer E.L. (2025). Your Contrastive Learning Problem Is Secretly a Distribution Alignment Problem. arXiv.

[28] Almousa O., Tashtoush Y., AlSobeh A., Zahariev P., Darwish O. (2026). SiAraSent: From Features to Deep Transformers for Large-Scale Arabic Sentiment Analysis. Big Data Cogn. Comput..

[29] Han W., Chen H., Poria S. (2021). Improving Multimodal Fusion with Hierarchical Mutual Information Maximization for Multimodal Sentiment Analysis. Proceedings of the 2021 Conference on Empirical Methods in Natural Language Processing.

[30] Pham H., Liang P.P., Manzini T., Morency L.P., Póczos B. (2019). Found in Translation: Learning Robust Joint Representations by Cyclic Translations Between Modalities. Proc. Aaai Conf. Artif. Intell..

[31] Tang J., Li K., Jin X., Cichocki A., Zhao Q., Kong W. (2021). CTFN: Hierarchical Learning for Multimodal Sentiment Analysis Using Coupled-Translation Fusion Network. Proceedings of the 59th Annual Meeting of the Association for Computational Linguistics and the 11th International Joint Conference on Natural Language Processing.

[32] Yu W., Xu H., Yuan Z., Wu J. (2021). Learning Modality-Specific Representations with Self-Supervised Multi-Task Learning for Multimodal Sentiment Analysis. Proc. Aaai Conf. Artif. Intell..

[33] Qian C., Xing S., Li S., Zhao Y., Tu Z. (2025). DecAlign: Hierarchical Cross-Modal Alignment for Decoupled Multimodal Representation Learning. arXiv.

[34] Li X., Zhang X., Miao D., Cheng X., Li D., Han H., Li Z. (2026). Mixture of Disentangled Experts with Missing Modalities for Robust Multimodal Sentiment Analysis. Inf. Fusion.

[35] Lin R., Hu H. (2022). Multimodal Contrastive Learning via Uni-Modal Coding and Cross-Modal Prediction for Multimodal Sentiment Analysis. Proceedings of the Findings of the Association for Computational Linguistics: EMNLP 2022.

[36] Lin Z., Liang B., Long Y., Dang Y., Yang M., Zhang M., Xu R. (2022). Modeling Intra- and Inter-Modal Relations: Hierarchical Graph Contrastive Learning for Multimodal Sentiment Analysis. Proceedings of the 29th International Conference on Computational Linguistics.

[37] AlSobeh A., Khamaiseh S., Middendorf D., Dziubinski J. CodeLLM-Auth: Multimodal Authorship Attribution and Verification via Supervised Contrastive Code Embeddings. Proceedings of the 2026 IEEE 50th Annual Computers, Software, and Applications Conference (COMPSAC).

[38] Xie Y., Zhu Z., Lu X., Huang Z., Xiong H. InfoEnh: Towards Multimodal Sentiment Analysis via Information Bottleneck Filter and Optimal Transport Alignment. Proceedings of the 2024 Joint International Conference on Computational Linguistics, Language Resources and Evaluation (LREC-COLING 2024).

[39] Li Y., Xing Y., Lan X., Li X., Chen H., Jiang D. AlignMamba: Enhancing Multimodal Mamba with Local and Global Cross-modal Alignment. Proceedings of the IEEE/CVF Conference on Computer Vision and Pattern Recognition (CVPR).

[40] He K., Chen B., Ding Y., Li F., Teng C., Ji D. (2026). PaSE: Prototype-aligned Calibration and Shapley-based Equilibrium for Multimodal Sentiment Analysis. Proc. Aaai Conf. Artif. Intell..

[41] Wang P., Zhou Q., Wu Y., Chen T., Hu J. (2024). DLF: Disentangled-Language-Focused Multimodal Sentiment Analysis. arXiv.

[42] Quan W., Feng Y., Zhou M., Zhao Y., Wang T., Yan D.M. (2024). TCAN: Text-oriented Cross Attention Network for Multimodal Sentiment Analysis. arXiv.

[43] Lei Y., Yang D., Li M., Wang S., Chen J., Zhang L. (2023). Text-oriented Modality Reinforcement Network for Multimodal Sentiment Analysis from Unaligned Multimodal Sequences. arXiv.

[44] Zadeh A., Liang P.P., Mazumder N., Poria S., Cambria E., Morency L.P. (2018). Memory Fusion Network for Multi-view Sequential Learning. Proc. Aaai Conf. Artif. Intell..

[45] Zhi Y., Li J., Wang H., Chen J. (2026). A Multimodal Sentiment Analysis Approach Based on Multiview Cross-Modal Fusion. IEEE Trans. Comput. Soc. Syst..

[46] Wang R., Wang Y., Cambria E., Fan X., Yu X., Huang Y., E X., Zhu X. (2025). Contrastive-Based Removal of Negative Information in Multimodal Emotion Analysis. Cogn. Comput..

[47] Zhang X., Wei W., Zou S. (2025). Modal Feature Optimization Network with Prompt for Multimodal Sentiment Analysis. Proceedings of the 31st International Conference on Computational Linguistics.

[48] Du Z., Wang Y., Yang X., Im S.K., Wang Z. (2026). MISA-GMC: An Enhanced Multimodal Sentiment Analysis Framework with Gated Fusion and Momentum Contrastive Modality Relationship Modeling. Mathematics.

[49] Lai S., Li J., Guo G., Hu X., Li Y., Tan Y., Song Z., Liu Y., Ren Z., Wang C. Shared and Private Information Learning in Multimodal Sentiment Analysis with Deep Modal Alignment and Self-supervised Multi-Task Learning. Proceedings of the 2024 International Joint Conference on Neural Networks (IJCNN).

